# Nanostructures for prevention, diagnosis, and treatment of viral respiratory infections: from influenza virus to SARS-CoV-2 variants

**DOI:** 10.1186/s12951-023-01938-8

**Published:** 2023-06-21

**Authors:** Esmaeel Sharifi, Satar Yousefiasl, Maria Trovato, Rossella Sartorius, Yasaman Esmaeili, Hamid Goodarzi, Matineh Ghomi, Ashkan Bigham, Farnaz Dabbagh Moghaddam, Maryam Heidarifard, Samiramis Pourmotabed, Ehsan Nazarzadeh Zare, Ana Cláudia Paiva-Santos, Navid Rabiee, Xiangdong Wang, Franklin R. Tay

**Affiliations:** 1 Department of Tissue Engineering and Biomaterials, School of Advanced Medical Sciences and Technologies, Hamadan University of Medical Sciences, Hamadan, 6517838736 Iran; 2 Dental Research Center, Dentistry Research Institute, Tehran University of Medical Sciences, Tehran, Iran; 3 Institute of Biochemistry and Cell Biology (IBBC), National Research Council (CNR), 80131 Naples, Italy; 4 School of Advanced Technologies in Medicine, Biosensor Research Center, Isfahan University of Medical Sciences, Isfahan, 8174673461 Iran; 5 Centre de recherche, Hôpital Maisonneuve-Rosemont, Montreal, QC Canada; 6 Départment d’Ophtalmologie, Université de Montréal, Montreal, QC Canada; 7 School of Chemistry, Damghan University, Damghan, 36716-45667 Iran; 8 Institute for Photonics and Nanotechnologies, National Research Council, Via Fosso del Cavaliere, 100, 00133 Rome, Italy; 9 Department of Emergency Medicine, School of Medicine, Hamadan University of Medical Sciences, Hamadan, 6517838736 Iran; 10 Department of Pharmaceutical Technology, Faculty of Pharmacy of the University of Coimbra, University of Coimbra, 3000-548 Coimbra, Portugal; 11 Group of Pharmaceutical Technology, Faculty of Pharmacy of the University of Coimbra, University of Coimbra, 3000-548 Coimbra, Portugal; 12 School of Engineering, Macquarie University, Sydney, NSW 2109 Australia; 13 Department of Materials Science and Engineering, Pohang University of Science and Technology (POSTECH), 77 Cheongam-ro, Nam-gu, Pohang, Gyeongbuk 37673 Republic of Korea; 14 Department of Pulmonary and Critical Care Medicine, Zhongshan Hospital, Fudan University Shanghai Medical College, Shanghai, 200032 China; 15 The Graduate School, Augusta University, Augusta, GA 30912 USA

**Keywords:** Antiviral therapy, Delta variant, Omicron variant, SARS-CoV-2, Viral infections

## Abstract

Viruses are a major cause of mortality and socio-economic downfall despite the plethora of biopharmaceuticals designed for their eradication. Conventional antiviral therapies are often ineffective. Live-attenuated vaccines can pose a safety risk due to the possibility of pathogen reversion, whereas inactivated viral vaccines and subunit vaccines do not generate robust and sustained immune responses. Recent studies have demonstrated the potential of strategies that combine nanotechnology concepts with the diagnosis, prevention, and treatment of viral infectious diseases. The present review provides a comprehensive introduction to the different strains of viruses involved in respiratory diseases and presents an overview of recent advances in the diagnosis and treatment of viral infections based on nanotechnology concepts and applications. Discussions in diagnostic/therapeutic nanotechnology-based approaches will be focused on H1N1 influenza, respiratory syncytial virus, human parainfluenza virus type 3 infections, as well as COVID-19 infections caused by the SARS-CoV-2 virus Delta variant and new emerging Omicron variant.

**Graphical Abstract:**

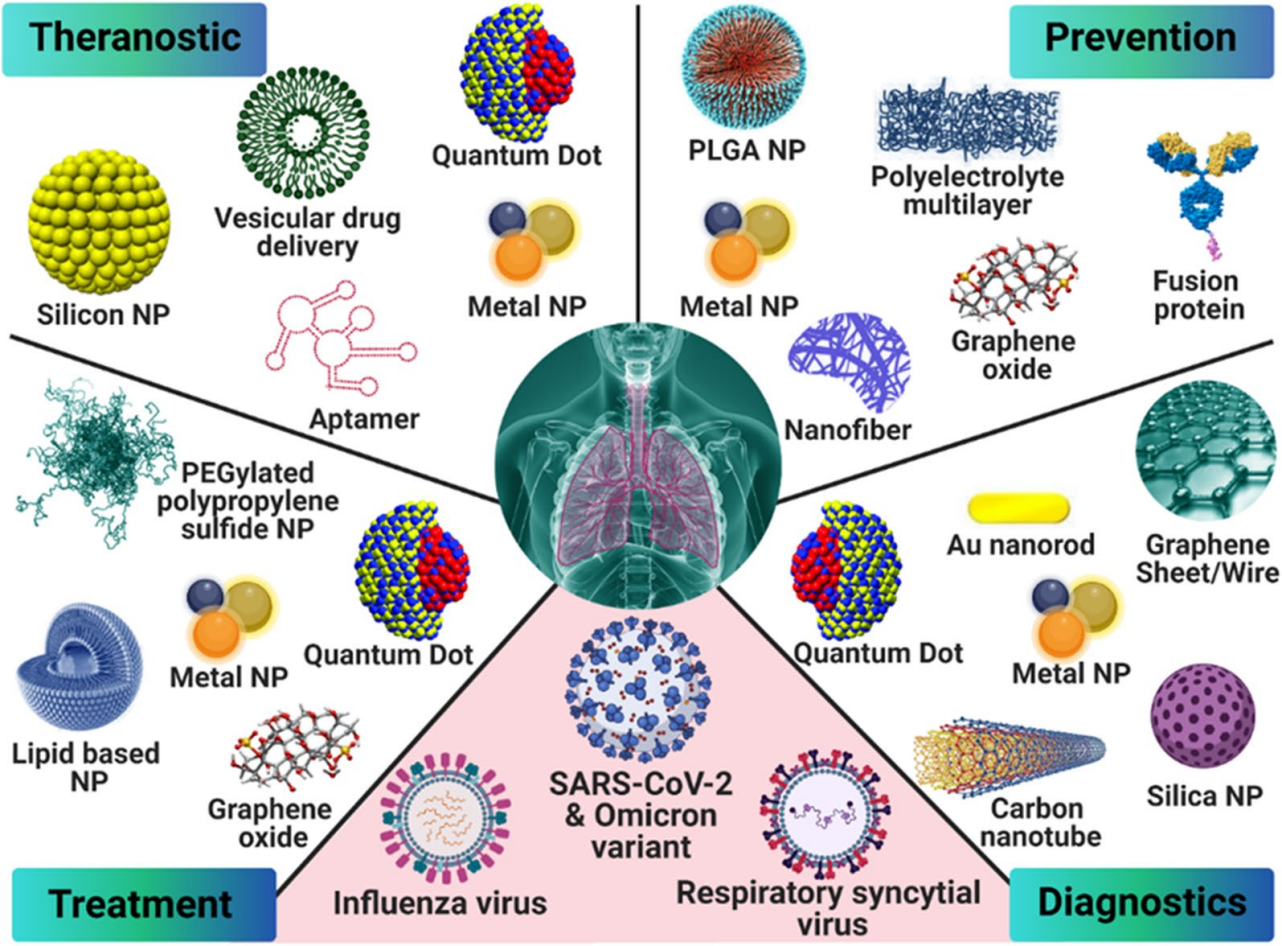

## Introduction

Viruses, often referred to as “organisms at the edge of life”, are responsible for some of the most severe pandemics and epidemics in the history of humankind. Viruses that are responsible for these catastrophic episodes include the dengue virus (DENV; 1779), human immunodeficiency virus (HIV; 1981), H1N1 influenza virus (1918), severe acute respiratory syndrome coronavirus (SARS-CoV; 2002–2003), swine flu (2009–2010), middle east respiratory syndrome coronavirus (MERS-CoV; 2012), Ebola virus (2013) and severe acute respiratory syndrome coronavirus 2 (SARS-CoV-2; late 2019) [1]. The most important reasons for the global rising in “coronavirus disease 2019” (COVID-19) cases were the unexpected origin of SARS-CoV-2, the lack of technologies to mitigate and decrease the spread of the virus, including its detection, testing, and prevention, as well as the unwillingness of the population at large to comply with wearing masks [2].

To date, the most challenging issue is addressing the health impact of SARS-CoV-2 in the global human community. The only solution to develop protective immunity is the formulation of vaccines to prevent and/or suppress virus infection and transmission [2]. In the meantime, the world has to rely on social preventive measures, processing procedures, and, most importantly, past experience acquired in dealing with analogous viral outbreaks such as SARS-CoV and MERS-CoV [3]. In this regard, the successful adaptation of biotechnology and nanomaterial-based vaccines can motivate researchers to investigate the use of a vast library of well-studied nanoparticles (NPs) against different viruses and the diseases they cause [4]. Nanotechnology has brought many benefits to the field of antiviral research which has the potential to overcome the limitations of conventional therapies. This emerging field improves the delivery of water-insoluble drugs; lengthens the time that drugs circulate in the body; enables the co-delivery of drugs; increases drug utilization effectiveness and decreases adverse effects via focusing on antibody alteration; preserves DNA and mRNA vaccines, removing obstacles for in vivo purposes; and nanomaterials’ physical characteristics can be used directly toward viruses [5, 6].

Nanoparticles such as metallic NPs [7–10], carbon nanotubes [11], polymeric NPs [12–14], and graphene [2, 15] have been used in biomedical applications especially experimentally for virus diagnosis, biosensor construction, and therapeutics against specific viruses that cause respiratory diseases [13, 16]. Some of these NPs may also be used for vaccine production or to deliver antiviral drugs to specific organs or tissues [17]. Some nanoparticles are capable of activating innate and adaptive immunity. For the administration of vaccines via the mucosa, nanoparticle size is a crucial factor because of the difficulty in penetrating the mucosal barrier [18]. Various respiratory diseases have been the targets for different types of NP-based vaccines—H1N1 influenza (poly-gamma-glutamic acid, chitosan, ferritin, Au), respiratory syncytial virus (polyanhydrides), and human parainfluenza virus type 3(oligomannose-coated liposome and poly (lactic-co-glycolic acid) [19, 20]. The use of NPs as probes for rapid, facile, and label-free detection of nucleic acids, proteins, and viral particles has advantages over conventional approaches in terms of high performance, sensitivity, specificity, stability, and size [21]. Recently, metallic NPs such as anti-spike antibody-attached AuNPs [22] and thiol-modified antisense oligonucleotides-capped AuNPs have been used for the development of experimental biosensors for identifying the sequences of SARS-CoV-2 by naked-eye screenings [23]. In vivo and in vitro studies have confirmed the potential of silver NPs upon inhalation, oral and dermal injection for the treatment of SARS-CoV-2-related disease (i.e., COVID-19) [24, 25].

The present review archives the applications of nanostructures for the prevention, diagnosis, and treatment of viral respiratory infections, including those caused by SARS-CoV-2 and its Alpha (B.1.1.7), Beta (B.1.351, B.1.351.2, B.1.351.3), Delta (B.1.617.2, AY.1, AY.2, AY.3) and Gamma (P.1, P.1.1, P.1.2) variants. Additional perspectives on the Omicron variant of SARS-CoV-2, H1N1 influenza virus, respiratory syncytial virus, and human parainfluenza virus type 3 will also be described.

## Respiratory viruses: an overview

The influenza virus is a member of the Orthomyxoviridae family with a negative-sense single-stranded RNA (ssRNA) genome that is consistently threatening to human health. The virus predominantly spreads by respiratory droplets and it can be transmitted via close contact with infected individuals. There are three types of influenza viruses infecting humans: type A, type B, and type C. Influenza virus may evolve through small changes that occur continuously in the viral surface proteins during viral replication or by major changes due to genetic re-assortments that introduce novel genes from the zoonotic reservoirs into the human virome. Such changes can eventually give rise to new influenza viruses that can potentially cause pandemics [26, 27]. Influenza virus is an enveloped virion containing two immunodominant glycoproteins, hemagglutinin, and neuraminidase. Hemagglutinin induces attachment of the virus to the host cells through its interaction with sialic acids on the surface of the target cell, driving the viral entry. Neuraminidase cleaves the sialic acids of glycoproteins at the cell and/or viral surfaces, allowing the release of viral progeny and preventing viral aggregation [28].

Respiratory syncytial virus (RSV) is a common respiratory virus that infects ciliated epithelial cells of the respiratory tract. RSV usually causes mild symptoms similar to a seasonal cold. However, in infants, elderly and immunocompromised individuals, RSV may affect the lower respiratory tract and the lungs, causing bronchiolitis and pneumonia, with abundant neutrophil infiltration and abnormal production of mucus that is responsible for airway obstruction, especially in infants [29]. RSV is a member of the Paramyxoviridae family, with a non-segmented negative-sense ssRNA genome encoding 11 proteins. Three proteins form the viral envelope: the glycoprotein (G) and fusion (F) transmembrane proteins, involved in the binding and fusion of the virion to the host cell, and a small hydrophobic protein (SH). RSV is classified into two antigenic groups, A and B, based on the protein G sequence. RSV infections cause only partial immunity, and reinfections are frequent in people of all ages. RSV has developed several mechanisms to counteract host immunity. The viral structural proteins contain extremely variable or highly glycosylated regions to limit the recognition of the host antibodies. Nonstructural-1 and -2 (NS1 and NS2) proteins (nsps) modulate innate and adaptive immune responses to RSV through inhibition of interferon (IFN) signaling, dendritic cell maturation, and T cell responses [30]. There is an urgent need to identify vaccination strategies to prevent RSV infections, especially in high-risk populations.

Coronaviruses (CoVs) are enveloped positive-sense ssRNA viruses with the largest genome among RNA viruses (up to 32 kilobases in size). They belong to the order Nidovirales, suborder Coronavirineae, family Coronaviridae, and subfamily Orthocoronavirinae. This subfamily is subdivided into four genera: *Alphacoronavirus* and *Betacoronavirus* (α- and β-CoV), infecting mammals, and *Gammacoronavirus* and *Deltacoronavirus* (γ- and δ-CoV), primarily infecting avian species [31]. While most human CoVs (hCoVs) cause relatively mild infections of the upper respiratory tract (common cold), three of the β-CoVs of zoonotic origins, namely SARS-CoV (SARS-CoV-1), MERS-CoV and SARS-CoV-2, caused severe respiratory disease outbreaks that occurred in China in 2002–2003, Saudi Arabia in 2012 and Wuhan (China) in late 2019, respectively [32].

About two-thirds of the CoV genome length is occupied by two large open reading frames (ORFs; ORF1a and ORF1b) that encode 15–16 nsps. In the other one-third of the genome, other ORFs encode at least four major structural proteins, including the trimeric spike (S), nucleocapsid (N), membrane (M), envelope (E) proteins, and different accessory proteins (APs) [33–36]. Some β-CoVs also express the membrane-anchored hemagglutinin-esterase (HE) protein [37]. The S protein is the major glycoprotein forming peplomers on the viral surface and is responsible for viral attachment, fusion, and entry into target cells [38]. The protein consists of S1 and S2 subunits. The S1 subunit contains an N-terminal domain and a receptor-binding domain (RBD) that is referred to as the C-terminal domain. Coronavirus infection is initiated when the S protein binds to a host receptor *via* RBD in the S1 subunit and initiates viral cell membrane fusion through the S2 subunit [39]. Angiotensin-converting enzyme 2 (ACE2) has been identified as the cell entry receptor for SARS-CoV-1 and SARS-CoV-2, while the MERS-CoV S protein engages dipeptidyl peptidase-4 (DPP-4, a.k.a. CD26) receptor to mediate viral entry. Recent bioinformatics approaches revealed the high potential affinity of SARS-CoV-2 S RBD to DDP-4, with RBD E484 residue being critical for DPP-4 binding [40]. Neuropilin-1 and CD147 function as host factors for SARS-CoV-2 infection, potentially enhancing viral entry by endocytosis [41, 42]. Integrins may also act as alternative receptors for SARS-CoV-2 entry into target cells [43].

**Fig. 1:**
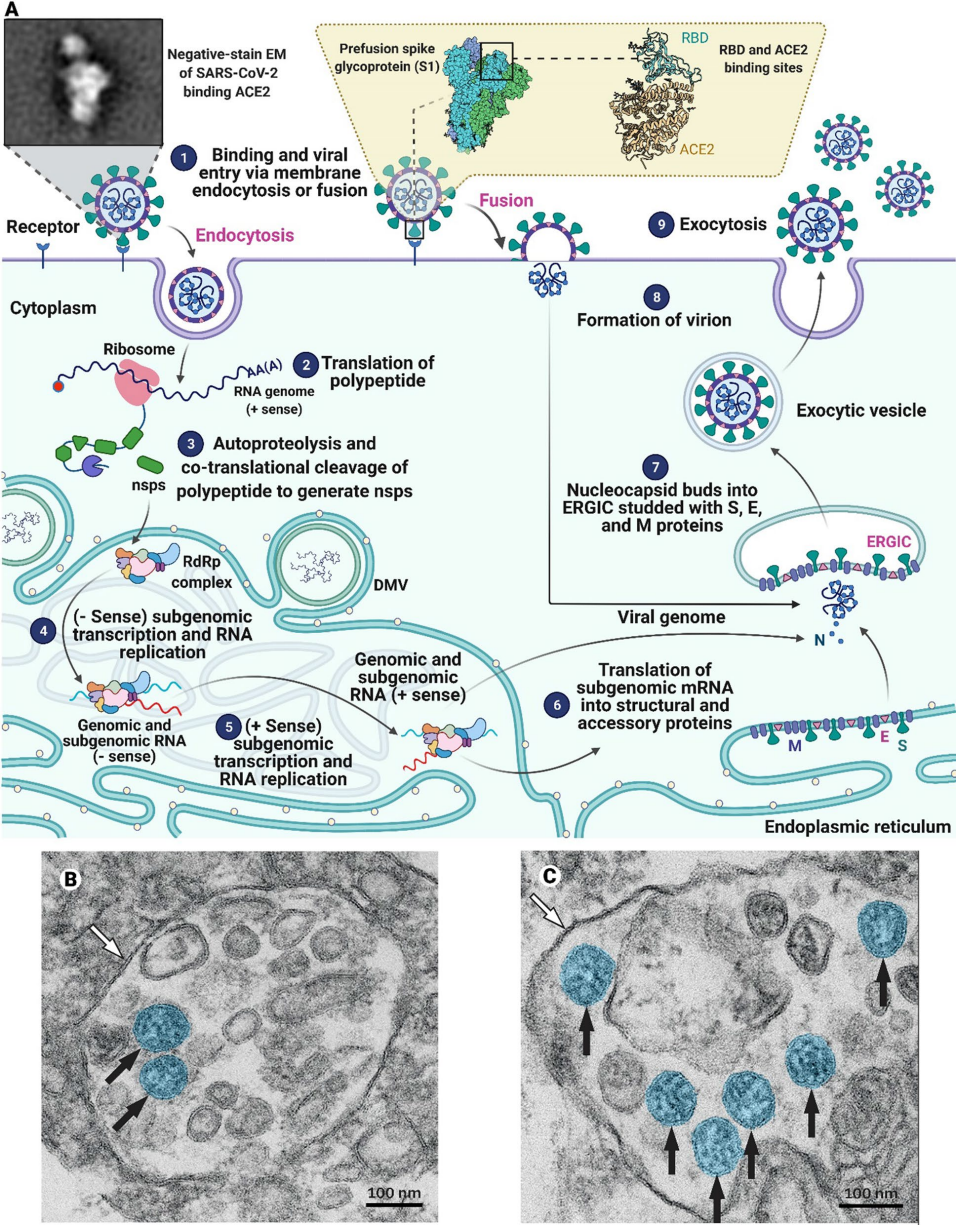
Mechanism of coronavirus (CoV) entry into target cells. **A** Coronaviruses bind to receptors on the host cell’s surface and enter the cell through endocytosis or fusion (1). The virus RNA genome is translated (2) to make polyproteins that are simultaneously broken down by proteases encoded in the polyprotein to produce RdRp complex components (3). In the following steps, (−Sense) subgenomic transcription and RNA replication (4) and (+Sense) subgenomic transcription and RNA replication (5) happen. The subgenomic mRNAs are translated into structural and accessory proteins (6). The positive-sense genomic RNA is bound by nucleocapsid and buds into the ERGIC studded with S, E, and M proteins (steps 6 and 7). The enveloped virion is exported from the cell by exocytosis (steps 8 and 9). SARS-CoV-2 particles in a **B** endothelial cell and a **C** type II pneumocyte at high magnification acquired by electron microscopy (black arrows refer to well-preserved coronavirus) (Parts **B** and **C** are reproduced from [51] with permission from Elsevier)

ACE2 is heterogeneously expressed in the human respiratory tract, being highest within the sinonasal cavity and pulmonary alveoli [44]. DPP-4 has been detected in the lung, and it is also widely expressed in epithelial cells of the kidney, liver, intestine, thymus, and bone marrow [45]. Apart from the engagement of functional receptors, proteolytic cleavage of CoV S protein at the S1/S2 boundary and ‘S2’ site by host cell proteases is essential to promote successful conformational changes in S that lead to viral fusion at the cellular or endosomal membrane. SARS-CoV and MERS-CoV employ the cell surface transmembrane serine protease 2 (TMPRSS2) and the endosomal cysteine cathepsin B and L (CatB/L) proteases for S protein priming/activation and entry [46, 47]. The TMPRSS2 and lysosomal cathepsins also activate the SARS-CoV-2 S protein and play an essential role in SARS-CoV-2 cell entry [48–50]. Depending on protease availability on the plasma membrane, CoV can enter target cells upon engagement of host receptor either by the “early pathway” or the “late pathway” [38]. The virus fuses with the cell membrane or endosomal membrane, releasing the viral genome into the cytosol (Fig. 1).

**Fig. 2:**
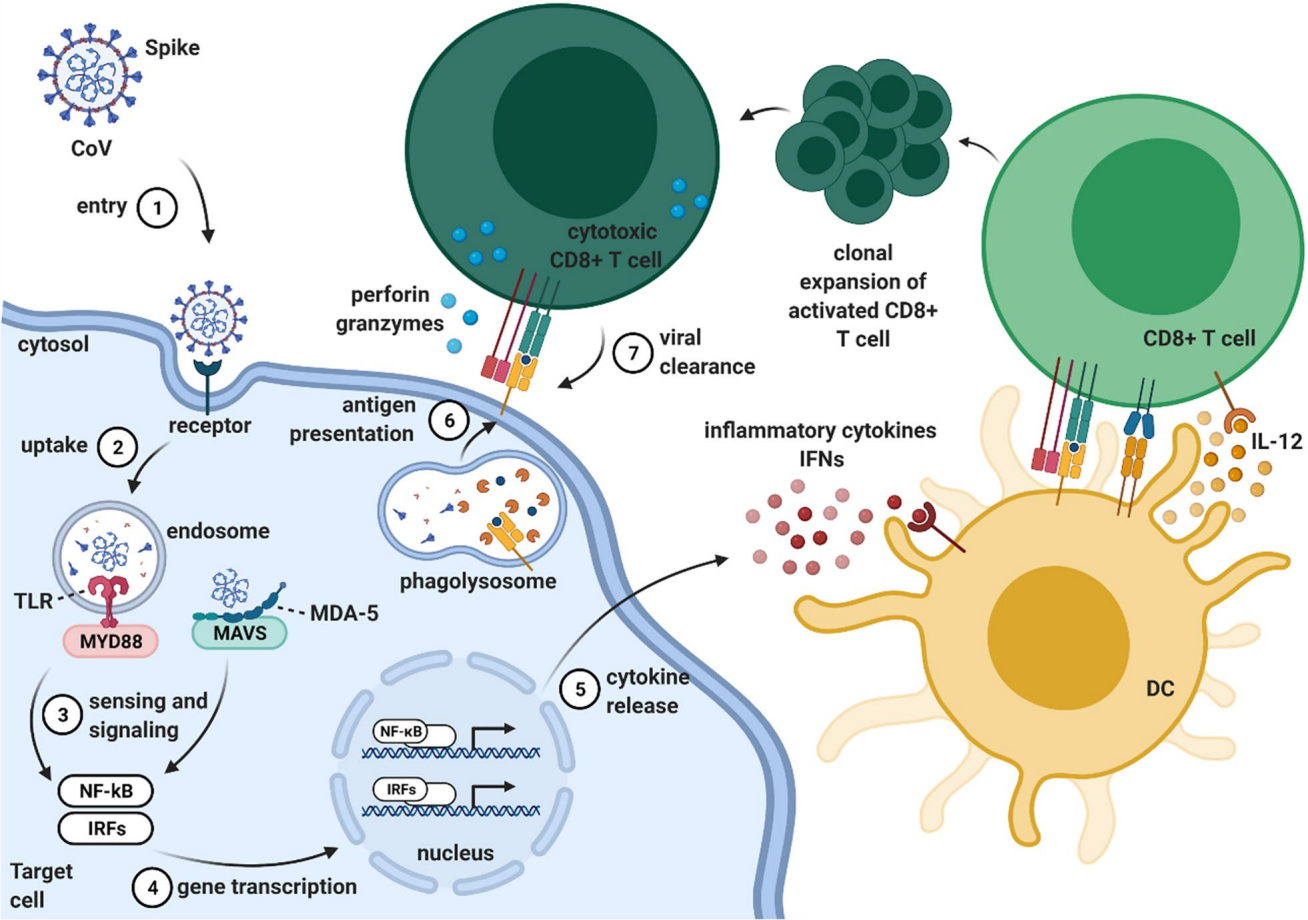
Host immune response to coronaviruses (CoVs): a schematic overview. Upon entry into the respiratory epithelium (1), CoV S protein binds to epithelial cells through the entry receptor, promoting viral uptake within the endosome (2). SARS-CoV-1, MERS-CoV, and SARS-CoV-2 are all sensed by the endosomal single-stranded (ss) RNA sensor TLR7; within the cytosol, MERS-CoV is detected by the cytosolic double-stranded (ds) RNA sensor MDA-5. The intracellular sensors respectively recruit the adaptor proteins MyD88 and MAVS and signal (3) for the activation of NF-κB and phosphorylation of IRF3/7 transcription factors (4). This results in the production of proinflammatory cytokines (e.g. IL-6 and TNF-α) and type I IFNs (IFN-α and IFN-β) (5). Type I IFNs recruit and stimulate immune cells, such as alveolar macrophages and lung-residing dendritic cells, to trigger innate and adaptive immune responses. Dendritic cells capture and process the viral antigen for presentation by MHC molecules to T cells. Clonally-expanded specific CD8^+^ T cells assist in clearing the infected cell, recognizing the viral peptide on MHC I (6/7). *DC* dendritic cell, *TLR* Toll-like receptor, *MDA-5* melanoma differentiation-associated protein 5, *MyD88* myeloid differentiation primary response 88, *MAVS* mitochondrial antiviral-signaling protein, *NF-κB* nuclear factor kappa-light-chain-enhancer of activated B cells, *IRF* interferon regulatory factor, *TNF* tumor necrosis factor, *IFN* interferon, *MHC* major histocompatibility complex. Created with BioRender.com

During the intracellular life cycle, CoVs express and replicate their genome to create new infecting CoV virions [52]. After CoV infection, viral RNA can be sensed and detected by pattern-recognition receptors (PRRs), including Toll-like receptors (TLRs), a retinoic acid-inducible gene I (RIG-I)-like receptors (RLRs), and nucleotide-binding oligomerization domain (NOD)-like receptors (NLRs) [53]. Signaling mediated by TLRs and RLRs leads to the production of type I IFNs and proinflammatory cytokines that recruit immune cells to trigger innate and adaptive immunity (Fig. 2) [35, 54, 55].

Coronavirus infection can additionally trigger the assembly and activation of NLR family pyrin domain containing 3 (NLRP3) inflammasomes to activate *caspase-1* and drive secretion of IL-1β and IL-18 proinflammatory cytokines. SARS-CoV, MERS-CoV, and SARS-CoV-2 also activate *caspase-8* and *caspase-3* to initiate PANoptosis, a unique inflammatory cell death pathway [54]. The exacerbated systemic inflammatory response contributes to a cytokine storm that causes severe inflammation, tissue damage, and acute respiratory distress syndrome (ARDS), eventually leading to death [56].

Viruses continuously change through mutations during periods of high spread. During the recent COVID-19 pandemic, SARS-CoV-2 genetic variants began to emerge at the end of 2020 and spread rapidly around the world. The Alpha (B.1.1.7), Beta (B.1.351, B.1.351.2, B.1.351.3), Delta (B.1.617.2, AY.1, AY.2, AY.3), Gamma (P.1, P.1.1, P.1.2), and Omicron (B.1.1.529) variants of SARS-CoV-2 are currently classified as variants of concern (VOC) [57]. Among these, the Delta variant showed higher infectivity than the parental virus, quickly succeeding in replacing the previously dominant variants. Several hypotheses have been formulated to explain the rapid propagation of the Delta variant. Among them, the presence of L452R and T478K mutations in S RBD has been suggested as features that improve ACE2 receptor engagement [58]. In addition, the P681R substitution in the furin cleavage site may be involved in facilitating the S1/S2 cleavage, thereby enhancing viral fusion [59]. More recently, mutations involving structural changes in the S trimer of the Delta variant have been suggested to be responsible for more efficient interaction with ACE2, even in cells with very low receptor levels, improving the infectivity of the B.1.617.2 strain [60]. Apart from the higher contagiousness, the Delta variant showed resistance to neutralization by some anti-S N-terminal domain (anti-NTD) and anti-S RBD (anti-RBD) monoclonal antibodies, as well as to antibodies present in the sera derived from convalescent COVID-19 patients [61].

The Omicron variant was first identified in South Africa and Botswana in 2021, and then subsequently spread to other regions of the world. This variant has multiple mutations, mainly located in the S protein, and still utilizes ACE2 as an entry receptor, but seems to be partially independent of TMPRSS2 [62]. Vaccination is still a promising approach for neutralization of the Omicron variant, although it has the capacity to escape neutralization by the *Pfizer* vaccine, albeit incompletely [63]. It is worth mentioning that the Omicron variant has a higher capacity for growth in the bronchus compared to the Delta variant, while its growth in the lung parenchyma is lower [64]. The reproduction number of the Omicron variant appears to be above 3, which can explain its high spread and more prominent infection in humans, compared to the Delta variant [65].

Although the COVID-19 vaccines currently in use are effective in mitigating the severe symptoms of SARS-CoV-2 infection, the emergence of VOCs and the occurrence of breakthrough infections indicate an urgent need for new diagnostic and therapeutic tools for the prevention and treatment of the disease.

## Prevention of viral respiratory infections

This section introduces two preventative measures for viral respiratory infections: prevention through disinfectant surface coatings and prevention with nanostructure-based vaccine formulations.

### Prevention through disinfectant surface coatings

The most probable way of catching a respiratory virus is by being in direct contact with an infected individual or touching contaminated surfaces on which the virus had spread [66]. Alcohol-based sanitizers (alcohol > 60%) are effective at eradicating some viruses, irrespective of the sanitizer formulation [67]. However, they are a temporary solution as surfaces need to be re-sanitized after alcohol evaporation. Therefore, developing substrates and surface coatings capable of inactivating viral pathogens is critical for the disinfection of high-touch surfaces in public transportation systems and healthcare centers.

**Fig. 3:**
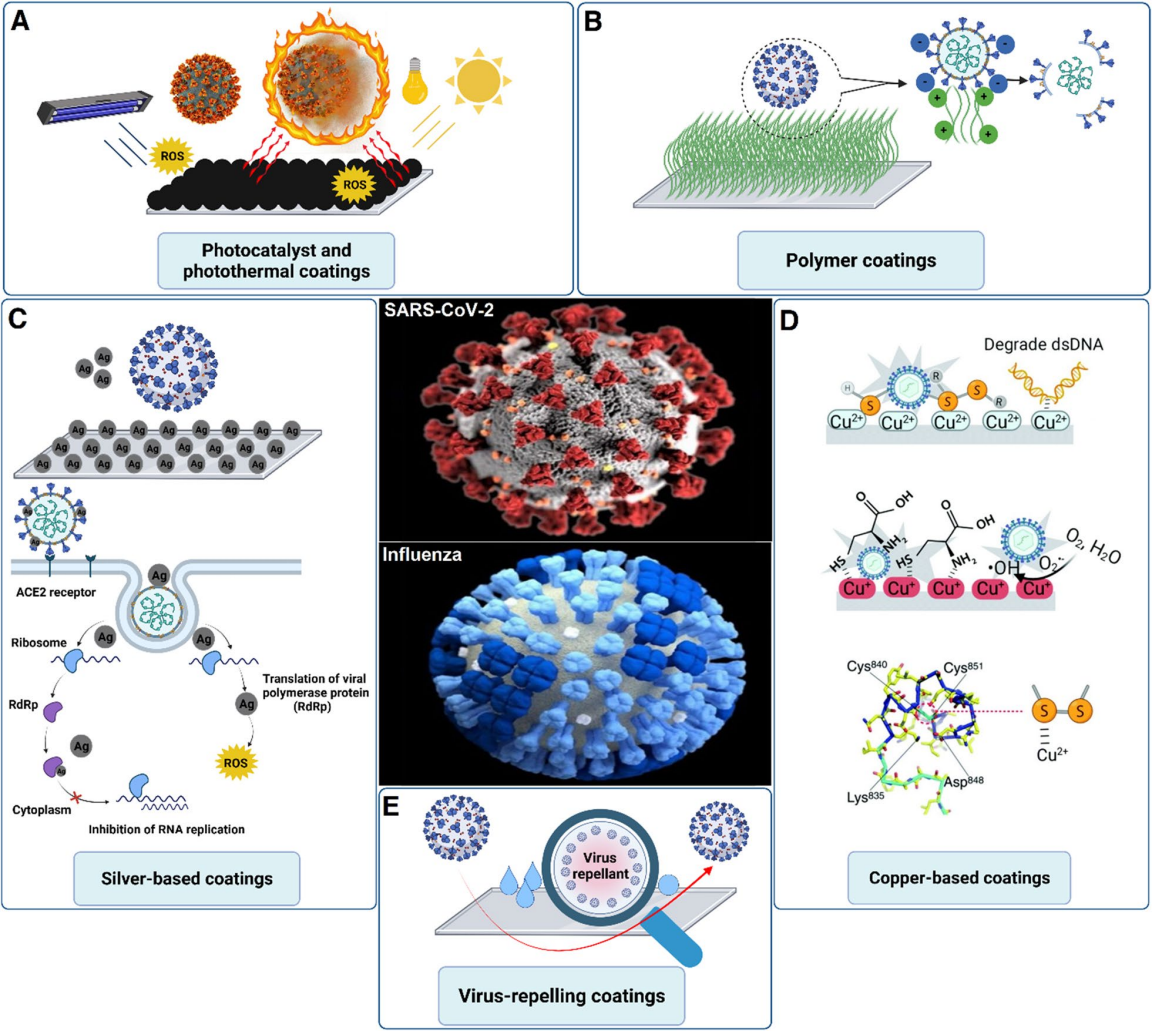
Antiviral coatings. Different surface modification approaches for developing antiviral surface coatings. These approaches include **A** photo-responsive reactive oxygen species (ROS) generation and hyperthermia; **B** inorganic polymer coatings; **C** silver coatings and the potential mechanism for inactivating SARS-CoV-2, **D** copper coatings and the potential interactions with SARS-CoV-2; **E** virus-repellant coatings over different substrates (Part **D** was reprinted from [70] with permission from RSC)

From an industrial point of view, the main reason for surface modification is to improve the corrosion and/or mechanical resistance of the substrate [68]. The idea is to modify the outermost layer of the substrate, which is in direct contact with the surrounding environment. With respect to modifications to obtain an antiviral surface, two approaches have been used. The first approach is based on surface coating with antiviral compounds (e.g., polymer, metal, inorganic, and composite materials). The other approach is to make the surface pathogen-repellant [69]. The antiviral modifications on different substrates will be covered in this section. Figure 3 summarizes the different surface coatings that may be applied on substrates to generate antiviral surfaces.

**Fig. 4:**
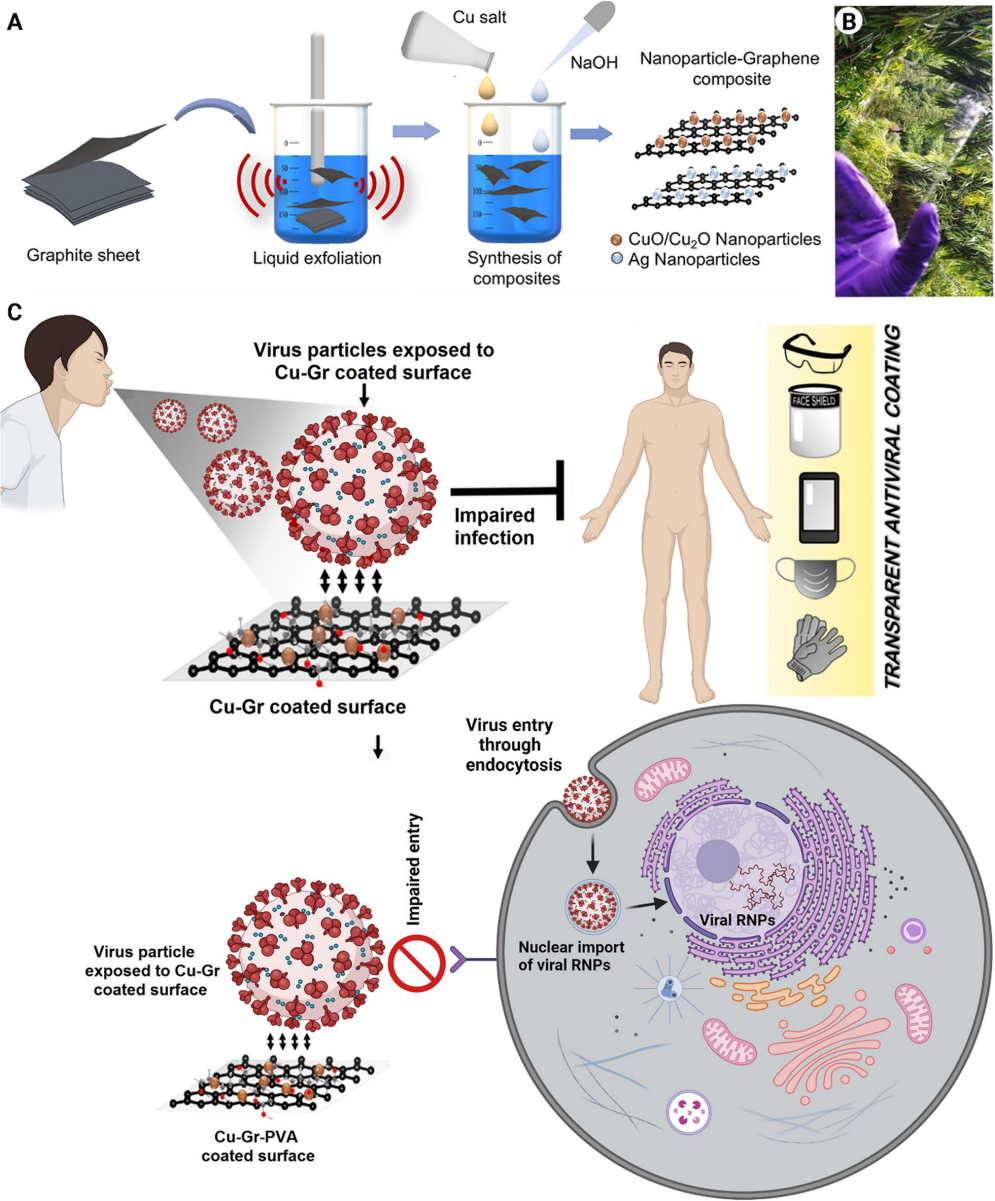
Copper oxide-graphite sheets (Cu-Gr) nanocomposite for antiviral applications. **A** Schematic representation of the liquid exfoliation of graphite and synthesis of metal–graphene nanocomposites. **B** A photograph of the coated nanocomposite on a glass sheet shows the transparency of the tempered mobile screen. **C** The proposed mechanism of the antiviral activity of Cu-Gr nanocomposite coating and its potential applications. *RNPs* ribonucleoproteins (Part **A** and **B** reprinted from [78] with permission from the American Chemical Society)

Copper has valuable properties that are utilized in medicine. These properties include angiogenesis, anticancer activity, antifungal potential, anti-inflammation, and antiviral activity [71–73]. Copper can target the viral genome, specifically the genes that are the origin of the virus infectivity. Similar to the antibacterial activity of copper, the generated reactive oxygen species (ROS) can damage the viral envelope or capsid. The damage is irreversible because viruses do not possess the repair mechanisms exhibited by bacteria or fungi [74]. Researchers have capitalized on this advantage to develop Cu-based antiviral surfaces. Indeed, Cu-alloys have been proposed for disinfecting a surface from human coronavirus 229E (HuCoV-229E) and turned out to inactivate 10^3^ plaque-forming units of HuCoV-229E on a 1 cm^2^ surface within 60 min [75]. A more recent study focused on coating copper powder on touch surfaces using thermal and cold spray techniques. Coating at a velocity of 500–1000 m/s and a temperature of 150–400 °C (thermal spray technique) resulted in a high density and diffusivity of copper powder with superior antiviral activity compared to the cold spray technique [76]. Copper has also been used with polyurethane for the coating of different objects. The efficacy of the composite coating was tested multiple times for up to 13 days, and the coating preserved its mechanical integrity apart from antiviral activity [77]. A unique copper oxide-graphite sheet nanocomposite was recently reported as a strong antiviral coating with a great degree of transparency. The nanocomposite can inactivate the Influenza virus within 30 min preventing its entry into the host cell. The proposed mechanism is interesting because 2D graphene can interact with the viral lipid membrane followed by rupturing the viral envelope to inactivate the virus. The 2D structure functions as the substrate for housing the copper ions. Because of its high surface area and interaction with viral particles, the nanocomposite is capable of trapping viruses. The trapped viruses that are in close vicinity with the copper ions are deactivated by those ions, resulting in the loss of their infectivity [78]. The production of the nanocomposite, its antiviral mechanism, and its potential applications are illustrated in Fig. 4.

Silver has always been at the epicenter of antimicrobial and antiviral applications [79]. The antiviral mechanism of silver nanoparticles involves the interaction of these nanoparticles with the viral membrane to prevent viral entry into host cells. Considering SARS-CoV-2, the S glycoprotein is targeted by silver nanoparticles that interfere with the binding to the host cell receptor. In addition, the release of silver ions from the nanoparticles decreases the pH of the respiratory epithelium, creating a harsh environment for the virus to survive [80]. Recently, a silver nanocluster-coated face mask has been developed with acceptable efficacy against SARS-CoV-2. The silver nanoclusters were coated in combination with silica through a sputtering technique, and the process may be effectively used for the coating of different substrates, including ceramics, polymers, metals, and composites. Such a technique enables disposable face masks to be used more than once, producing less waste [81].

Zinc is a multifunctional ion endowed with antibacterial and antiviral activities, regenerative potential, and biological functionality [82, 83]. The antiviral activity of zinc dates back to 1974, when its potential against the human rhinovirus was first reported [84]. Although the antiviral mechanism of zinc has not been elucidated yet, it has been reported that zinc exerts its antiviral activity by affecting physical processes such as virus attachment and by generating ROS [85, 86]. In the context of surface coating, zinc has been used in combination with other metal elements like copper and silver to reinforce the antiviral potential of the substrates [87]. Recently, a disinfectant spray composed of zinc (II) oxide nanoparticles was reported for SARS-CoV-2 treatment. The disinfection spray inhibited viral activity at a very low concentration with a half-maximal inhibitory concentration (IC50) of 526 ng/mL) [88].

A developed microplate from ZnO nanowire was made-up and coated with SARS-CoV-2 as a fluorescent immunoassay to detect antibodies specific to SARS-CoV-2 NP. The ZnO nanowire microplate bound high levels of SARS-CoV-2 nucleocapsid marked to histidine without any surface treatment. In addition, a novel serological assay based on the ZnO-nanowire microplate was more sensitive than a commercial immunoassay, enabling early detection of anti-SARS-CoV-2 nucleocapsid IgG antibodies in asymptomatic patients with COVID-19 [89]. In another study, immobilization of SARS-CoV-2 recombinant trimeric spike protein on zinc oxide nanorod-modified fluorine-doped tin oxide substrates was used as a biosensor for COVID-19 serology testing. No cross-reactivity with seasonal coronavirus was noticed via the ZnO nanorod immunosensor, and more fascinatingly, the sensor showed higher sensitivity once associated with negative enzyme-linked immunosorbent assay results [90].

The use of photocatalytic materials for antiviral applications has recently attracted attention because these materials eradicate viruses through the generation of ROS and/or heat when exposed to light [91]. Titanium oxide (TiO_2_) is an inorganic component that may be used as a potential disinfectant in surface coating applications [92]. Its photocatalytic properties result in the inactivation of bacteria and viruses [93]. The antimicrobial/antiviral activity of TiO_2_ is due to the light absorption followed by the generation of ROS, including superoxide anion and hydroxyl radicals [94]. A study that examined the photocatalytic-related antiviral property of TiO_2_ coating against the enveloped influenza virus reported a 3.6-log reduction in influenza virus activity after four hours of exposure to ultraviolet light [95]. A composite coating made of TiO_2_ in combination with other metal elements such as Cu and Ag was developed, with the hybridized composite exhibiting increased potent antiviral activity [96].

It is dubious how a photocatalytic compound, activated upon exposure to sunlight, may be used indoors with inadequate light. An attempt has been made to alter the TiO_2_ structure for improved antiviral applications to address this issue. A surface coating composed of fluorinated TiO_2_ was developed to combat several types of human norovirus, bacteriophage MS2, feline calicivirus, and murine norovirus. Modification of TiO_2_ with fluorine resulted in a surface coating that exhibited catalytic activity in the presence of ultraviolet light emitted by fluorescent light [97]. These surface disinfectants can be applied in places with no direct sunlight, such as metro stations. Nevertheless, more investigation is required. A multifunctional coating composed of silver and TiO_2_ was formed through the sol-gel method (dip-coating) on soda-lime glass substrates. The antibacterial and antiviral capabilities of the coating were evaluated using *Escherichia coli* and H1N1 virus. The combination of silver and TiO_2_ yielded a powerful disinfectant against both organisms, with nearly 100% activity [93].

Under the umbrella of photocatalyst materials, an antiviral surface coating against SARS-CoV-2 has been developed with both hydrophobicity and photocatalytic properties. The hydrophobic surface coating was obtained when a CO_2_ laser was applied in the presence of nitrogen. The application of sunlight-simulating xenon light (1 kW m^−2^) resulted in a temperature increase to 55 °C in just 10 s, with the final temperature reaching 62 °C. For Joule heating, the application of a direct current voltage of 7.5 V to a surface (10 × 10 cm^2^) resulted in a temperature increase of up to 50 °C. A temperature of 46 °C is enough for virus inactivation. The application of 0.5 kW m^−2^ of xenon light or a direct current power of 20 mW cm^−2^ can yield the temperature required for antiviral activity. These requirements are easily matched outdoor with low solar irradiation or indoor with 2–3 AAA batteries [69].

Organic surface coatings are another class of antiviral materials with great potential for scaling up because polymers can be easily coated over various types of substrates [98]. A subcategory of this family is composed of positively-charged polycations which have been used against different types of pathogens [98]. This type of coating provides a trap for negatively-charged viruses, causing them to be completely disintegrated. One of the prominent features of polycation surface coatings is their physical and chemical stability. The polycation surface coating is mainly accomplished through painting over a substrate. Even after several times of washing, the coating preserved its antiviral property [99]. Amine-rich polyethyleneimine is a polycation that has been extensively used as protein and virus absorbents [99, 100], often in combination with heavy metal ions to improve their complexation [101]. In 2011, commercial face masks were modified with polyethyleneimine, and the modified masks collected 99.9% of the sprayed viral suspension in the air [102]. Although polycation polymers are good candidates for antiviral activity, their antiviral activities are further potentiated when combined with hydrophobic materials. A hydrophobic cationic coating with improved antiviral activity was developed through the combination of polyethyleneimine and acetyl groups [103]. Another governing factor affecting the antiviral activity of polyethyleneimine is its molecular weight. Different molecular weights, including 2, 25, and 750 kDa, were tested, and 750 kDa was the only one that successfully deactivated the influenza virus. Interestingly, this study shed light on the effect of surface charge on the antiviral activity of polycation, polyanion, and neutral coatings. Although both the polycation and polyanion coatings demonstrated antiviral activity, a stronger activity was observed for the polycation coating because there were more positively charged domains to attract influenza virus. In contrast, the neutral surface functioned only as a non-virucidal coating [104]. Polycationic coatings might be very promising candidates for disinfecting SARS-CoV-2 contaminated high-touch surfaces owing to easy and cost-effective coating process. However, more modifications are required to improve their efficacy.

### Nanostructure-based vaccine formulations

**Fig. 5:**
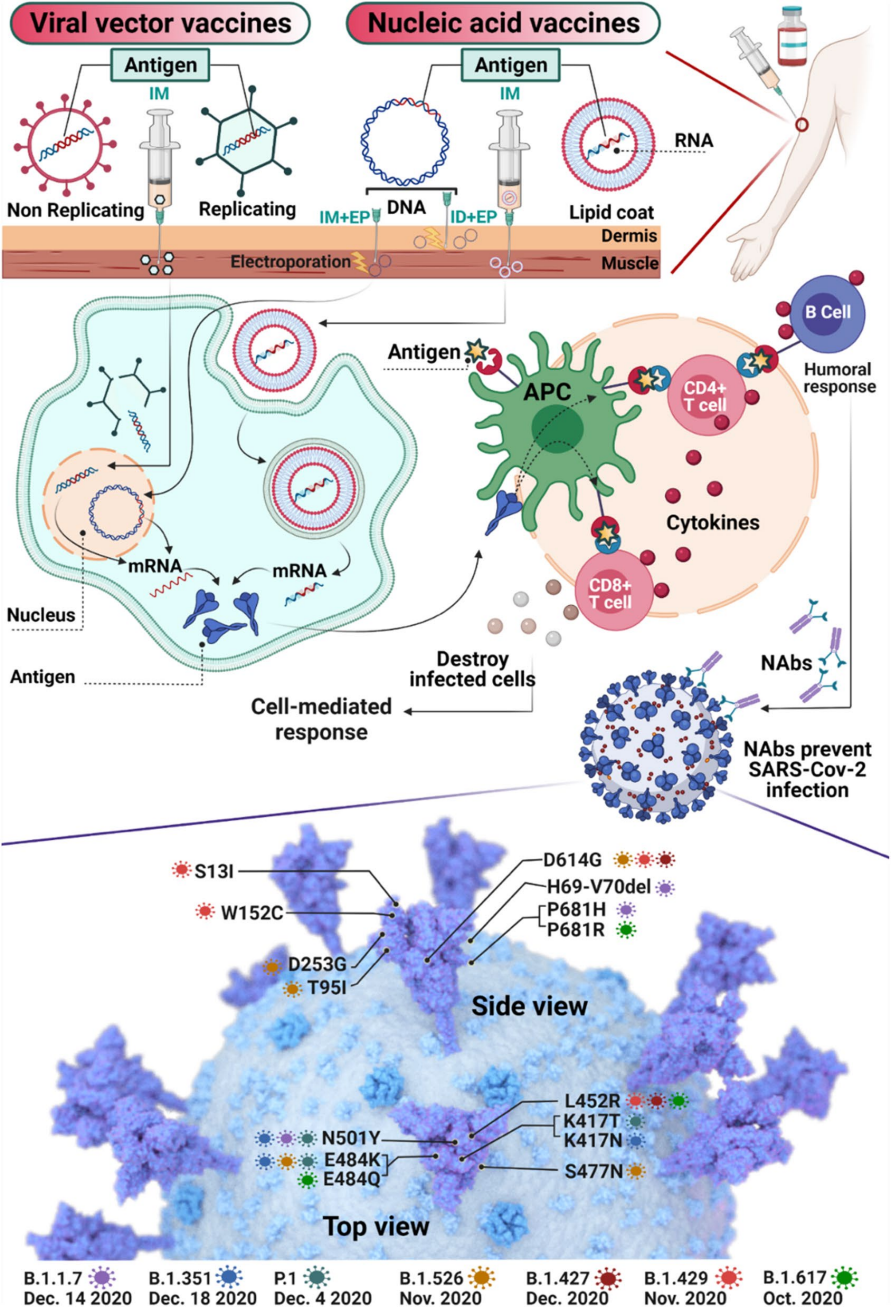
Gene-based vaccine candidates for SARS-CoV-2 including viral vector vaccines (non-replicating and replicating) and nucleic acid vaccines (DNA and RNA with a lipid coat). Viral vector vaccines enter the nucleus after IM injection. mRNA coated with lipids reaches the cytoplasm through endocytosis after IM injection. Eventually, mRNA translation in the cytoplasm leads to antigen expression. DNA-containing vaccines injected as IM + EP or ID + EP lead to gene transcription by entering the nucleus (EP involves the application of electrical pulses, generating pores in skin cells to enhance cellular uptake of genetic material), and translation in the cytoplasm. The produced antigens can be uptaken and presented by APCs, activating B cells, CD4+ T cells, CD8+ T cells and leading to the induction of humoral response with the production of neutralizing antibodies that can bind to the spike proteins on SARS-CoV-2 virus, preventing SARS-CoV2 infection, and/or the T-cell mediated destruction of infected cells. The magnification indicates a three-dimensional 3D rendering of SARS-CoV-2 that showcases the key spike protein mutations on each of the SARS-CoV-2 variants of concern: B.1.1.7, B.1.351, P.1, B.1.526, B.1.427 and B.1.429. *IM* intramuscular, *ID* intradermal, *EP* electroporation, *APC* antigen-presenting cell, *NAbs* neutralizing antibodies

Nanoparticles have been largely used as immunostimulatory candidates for the development of vaccines [105]. For example, ribonucleic acids conjugated to ferritin-based nanoparticles have been exploited as a vaccine against MERS-CoV [106]. During the COVID-19 pandemic, there was an urgent need for vaccine development to stop viral transmission. The vaccine candidates are based on different platforms: nucleic acids (mRNA and DNA, viral vectors, inactivated viruses, and protein vaccines [107]. The viral capsid or the lipid membrane serves as a barrier protecting the nucleic acid from degradation, and the nanoparticle functions as a carrier for mRNA delivery into cells. Cells can transcribe the viral DNA and translate the mRNA into the spike protein. The resulting antigenic determinants are presented to immune cells through the major histocompatibility complex I/II pathways to activate T cells (CD4+ and CD8+) and induce antibody production (Fig. 5) [108].

The *Pfizer-BioNTech* COVID-19 vaccine BNT162b2 (Pfizer, New York City, New York, USA; BioNTech, Mainz, Germany) comprises a lipid nanoparticle encapsulating a nucleoside-modified mRNA encoding the pre-fusion SARS-CoV-2 S protein [109]. mRNA is inherently unstable and is prone to self-hydrolyze in the presence of hydroxyl groups, and it is rapidly degraded by ribonucleases. Hence, in the BNT162b2 vaccine, the mRNA is embedded in lipid nanoparticles to increase its stability and ability to be taken up by cells. The lipids bilayer contains tertiary and quaternary amines suitable for electrochemical interaction with the anionic mRNA. Cholesterol is added to the lipid bilayer to increase its stability by mitigating the repulsive forces of the positively-charged lipids. Finally, polyethylene glycol (PEG)-modified lipids are used to render the lipid nanoparticles soluble [110]. These non-ionic PEGylated lipids help to stabilize the lipid nanoparticles colloidally. The PEGylated lipids also enable the vaccine to evade phagocytosis by the mononuclear phagocyte system [111].

Similarly, the *Moderna* COVID-19 vaccine (Moderna Inc., Cambridge, MA, USA) is made of mRNA coding for the pre-fusion spike protein. The mRNA is encapsulated in a lipid nanoparticle composed of phospholipids with positively-charged amine groups, cholesterol, and a PEG-based lipid that is adjuvanted with a lipid named SM-102. The *Moderna* COVID-19 vaccine encodes for the S-2P antigen, the full-length viral spike protein with two consecutive proline substitutions at amino acid positions 986 and 987 to stabilize the pre-fusion form of the protein for infecting host cells [112].

The *Novavax* COVID-19 vaccine (Novavax KInc., Gaithersburg, MD, USA) is a protein-based vaccine, which is currently approved for adults over 18. It is composed of the spike protein integrated on the surface of a nanoparticle. The formulation contains a saponin-based adjuvant (Matrix–M^™^). The nanoparticle is composed of polysorbate 80 (also known as tween 80). This fatty acid comprises a tail of oleic acid and a head of polyethoxylated sorbitan, making it a non-ionic micelle [113].

There have been in-vitro and in-vivo studies on mRNA, DNA, and protein-based vaccines. Regarding protein-based vaccines, the S RBD is the main target for developing a vaccine. The main difficulty in developing RBD-based vaccines is their low immunogenicity. Recently, linking a nanoparticle to the S RBD through a covalent bond has been proposed as a strategy to increase S immunogenicity. Kang et al. developed RBD-conjugated nanoparticles (RBD-ferritin, RBD-mi3, and RBD-I53-50 nanoparticles) through irreversible conjugation of recombinant proteins to nanoparticles by a SpyTag-SpyCatcher system. The efficacy of this fused construct was higher than the RBD alone. Vaccination with RBD-conjugated nanoparticles induced a high titer of neutralizing antibodies in mice. The neutralizing activity against the virus was 8 to 120-folds greater than that of the monomeric RBD [114].

RBD and heptad repeat (HR) conjugation to nanoparticles (ferritin) have been proposed to neutralize SARS-CoV-2. In a recent study, two vaccination strategies have been developed by using ferritin nanoparticles conjugated to RBD and RBD-HR. The RBD-HR nanoparticles induced robust neutralizing antibodies and a higher percentage of T follicular helper (Tfh) cells and B cells. Conjugation of nanoparticles to S RBD and HR may be a valuable strategy for future vaccine development [107]. Conjugation of viral particles through covalent bonds to nanostructures that enhance their immunogenicity is an essential concept in developing new vaccine strategies.

Self-amplifying mRNA (saRNA) encoding the spike protein and encapsulated in lipid nanoparticles has been employed to immunize mice. These saRNA-lipid nanoparticles produced a dose-dependent SARS-CoV-2 IgG antibody response with neutralizing activity, together with a cellular Th1-biased response in a preclinical murine model [115]. In addition, 5′ and 3′ untranslated regions of mRNA have been analyzed and optimized to enhance mRNA stability and translation. Modification of 5′ and 3′ untranslated regions of the RBD and S mRNA combined with its encapsulation in a lipid-derived nanoparticle *N*^1^,*N*^3^,*N*^5^-tris(3-(didodecylamino)propyl)benzene-1,3,5-tricarboxamide (TT3) induced strong antigen production and antigen-specific antibodies [116].

Nanostructures employed in vaccine formulations may take different roles as follows: (i) being an adjuvant to enhance the vaccine efficacy, (ii) being an antigen delivery system to improve antigen presentation to the immune system, (iii) or functioning as the main component of the vaccine formulation [117]. For instance, carbon nanotubes, carbon black nanoparticles, poly(lactic-glycolic acid) and polystyrene, titanium dioxide, silicon dioxide, and alumina nanoparticles can activate NLRP3-induced inflammation and enhance immune responses [118]. These nanoparticles cause lysosomal instability and ROS production by activation of specific signals, which are ultimately associated with the release of cathepsin B and cysteine ​​proteases [119]. Interleukins are then produced, which result in the activation of adaptive immune cells [120, 121]. In this context, chitosan nanocapsules delivering a model antigen on their surface and adjuvanted with an immunostimulating oily core could boost the immunological response and induce long-lasting immunity against the hepatitis B virus. The small size of nanoparticles allows them to be easily internalized and processed by the immune cells, thereby increasing the chance of antigen recognition and a better and longer immune response [122, 123].

Mineral and polymer nanoparticles, virus-like particles (VLPs), self-assembled protein nanoparticles, and liposomes have been identified as antigen carriers. One of the most significant attributes of nanoparticles is related to their protection of antigens against proteolytic degradation [124]. Another benefit of antigen encapsulation throughout a nanoparticle is an increase in the antigen storage time and shelf-life [125].

Nanoparticles easily penetrate capillaries and mucosal surfaces through subcutaneous and intramuscular injections, or oral and intranasal mucosal sites, respectively [126]. Subcutaneous or intramuscular injections provide systemic immunity. In contrast, mucosal vaccination (via mouth or nose) induces humoral and cellular immune responses at the mucosal level, thereby providing better protection against respiratory viruses [117].

**Fig. 6:**
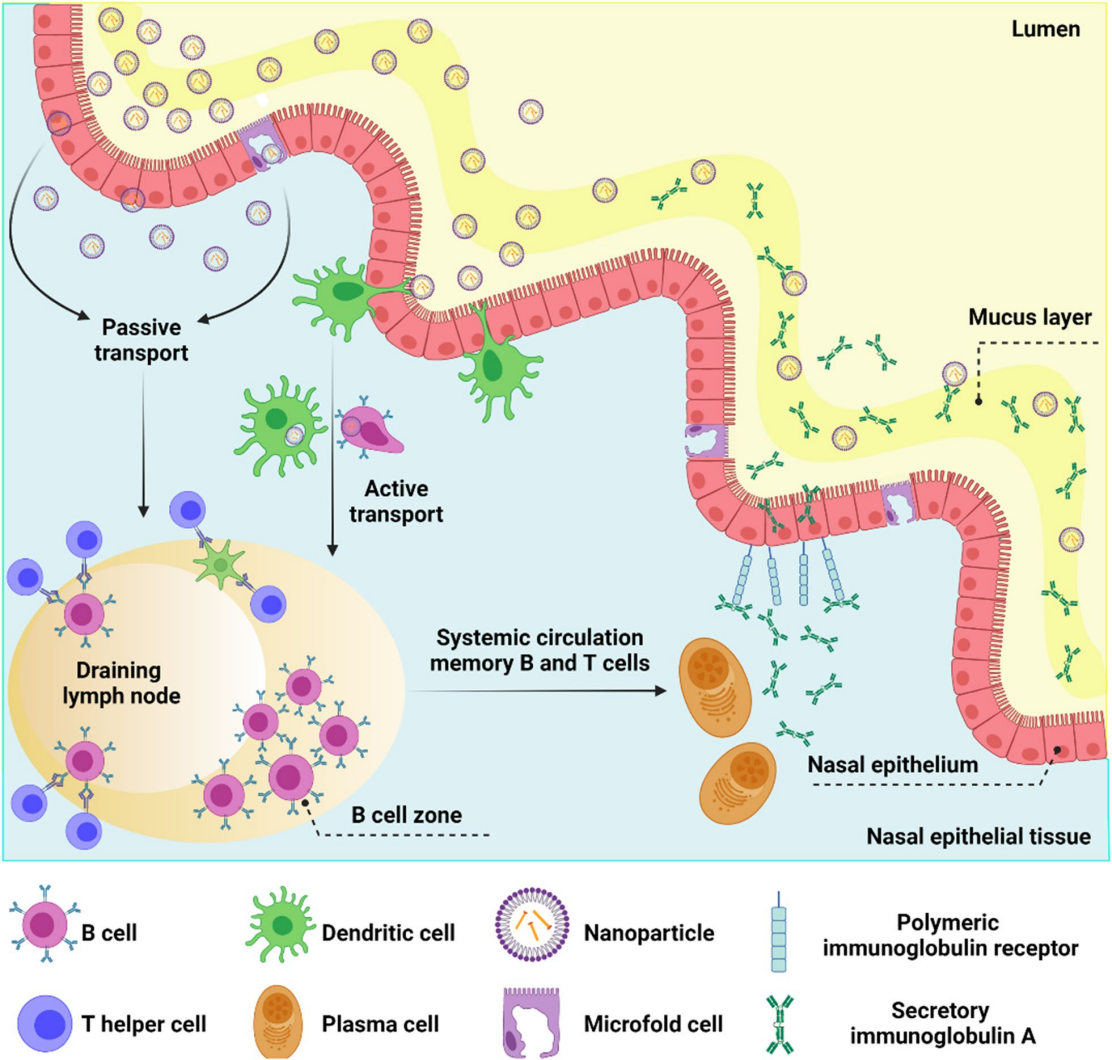
Nanoparticles traffic the nasal epithelium to induce immune responses. Nanoparticles can cross the mucosal respiratory layer to reach the nasal epithelial tissues through microfold cells (M cells). The M cells are antigen-delivering epithelial cells that transport antigens by transcytosis to the underlying immune cells. Nanoparticles can also be actively captured by dendritic cells or transmitted passively through epithelial cell junctions. Cells that have encountered nanoparticles migrate to the lymph nodes and activate T helper cells. Activated T helper cells induce B cell proliferation in the lymph nodes (B cell zone) and enter the systemic circulation to reach the site of inflammation. Among class-switched B cells, IgA + B cells differentiate into antibody-secreting plasma cells to produce IgA dimers. IgA dimers are transferred to the mucosal surface by the polymeric immunoglobulin receptor that translocates the secreted IgA across the epithelium

Respiratory viruses are transmitted from the upper respiratory tract to the lower respiratory tract by secretions containing viral particles [18]. Nasal connective tissue represents the first line of defense against respiratory viruses because it is the first site for the detection of inhaled antigens [127]. Nasal connective tissue consists of various narrow epithelial ducts and is a collection of lymphoid follicles (B-cell regions), inter-follicular regions (T-cell regions), macrophages, and dendritic cells [128]. Nano-vaccines can deliver antigens to nasal connective tissue by following a pathway similar to that chosen by respiratory viruses. The delivered antigens may be taken up by dendritic cells and macrophages to stimulate the immune system. Accordingly, formulation, size, and presentation of antigens are important aspects when designing nasal connective tissue-targeted nano-vaccines (Fig. 6) [129]. The use of surface modifications to enhance the selective binding of nanoparticles to specific immune receptors opens a plethora of options for activating the humoral and cellular responses [130]. Raghuwanshi and his colleagues demonstrated a twofold increase in antigen uptake by dendritic cells by using a multi-subunit approach. The latter consisted of an antigen encapsulated in biotinylated poly(lactic-co-glycolic acid) nanoparticles complexed to a bifunctional fusion protein. The protein was made of a core-streptavidin fused to a single-chain variable fragment of an antibody for dendritic cell targeting [131]. Similarly, Lynn et al. demonstrated that TLR-specific agonists bound to nanoparticle surfaces might be used as a mechanism for targeting lymph node-resident cells. This strategy enhanced antigen-specific immune responses and reduced systemic biodistribution to minimize side effects [132].

According to the WHO (https://www.who.int/publications/m/item/draft-landscape-of-covid-19-candidate-vaccines; assessed on march 30, 2023), 198 vaccines are now in preclinical stages, while almost 166 are in clinical development. Information on COVID-19 vaccines based on nanoparticles that are either in late-stage clinical studies (phase 4) or early-stage clinical trials (phase 3) is provided in Table 1.

**Table 1:** Information on the some of the nanoparticle-based SARS-CoV-2 vaccines that are in phase-4, phase-3 of clinical trials [133, 134]

Phase	Vaccine name	Type of vaccine	Developer
Phase 4	CoronaVac	Inactivated virus	Sinovac
Phase 4	Spikevax	RNA based vaccine	Moderna
Phase 4	BNT162b2	RNA based vaccine	Pfizer/BioNTech
Phase 4	Ad5-nCoV-IH	Viral vector (non-replicating)	CanSino Biological Inc./Beijing Institute of Biotechnology
Phase 4	Ad26.COV2.S	Viral vector (non-replicating)	Janssen Pharmaceutical
Phase 4	MVC-COV1901	Protein subunit	Medigen Vaccine Biologics
Phase 3	Nanovax	Recombinant Sars-CoV-2 Spike protein, Aluminum adjuvanted (Nanocovax)	Nanogen Pharmaceutical Biotechnology
Phase 3	SCB-2019	Protein subunit	Clover Biopharmaceuticals Inc./Dynavax
Phase 3	UB-612	Protein subunit	Vaxxinity
Phase 3	GBP510	Protein subunit	SK Bioscience Co., Ltd.

## Viral respiratory disease diagnostics

Diagnosis is a fundamental process in medicine that provides information to the physician to manage the medical issue properly. This highlights the importance of early diagnosis of viral diseases to prevent virus spread and curb the outbreak [135, 136]. Virus detection methods currently include cell culturing, serology methods based on specific viral antigens, or antibody detection using enzyme-linked immunosorbent assay (ELISA), immunofluorescence immunoperoxidase, and hemagglutination assay, as well as molecular methods such as nucleic acid detection by a polymerase chain reaction and gene sequencing [137, 138]. These conventional methods are time-consuming and not very accurate. The limitations associated with these methods emphasize the urgent need to develop more rapid, accurate, sensitive, and straightforward diagnostic methodologies [139].

Different nanomaterials have been employed for the diagnosis and tracking of pathogenic viruses. They are based on metallic nanoparticles (e.g., gold nanoparticles, silver nanoparticles, magnetic nanoparticles, copper nanoparticles, silica nanoparticles), carbon-based nanomaterials (e.g., carbon dots, graphene oxide, carbon nanotubes), and polymeric nanoparticles. Some of these nanomaterials-based biosensors will be further discussed in this section [140–144].

**Fig. 7:**
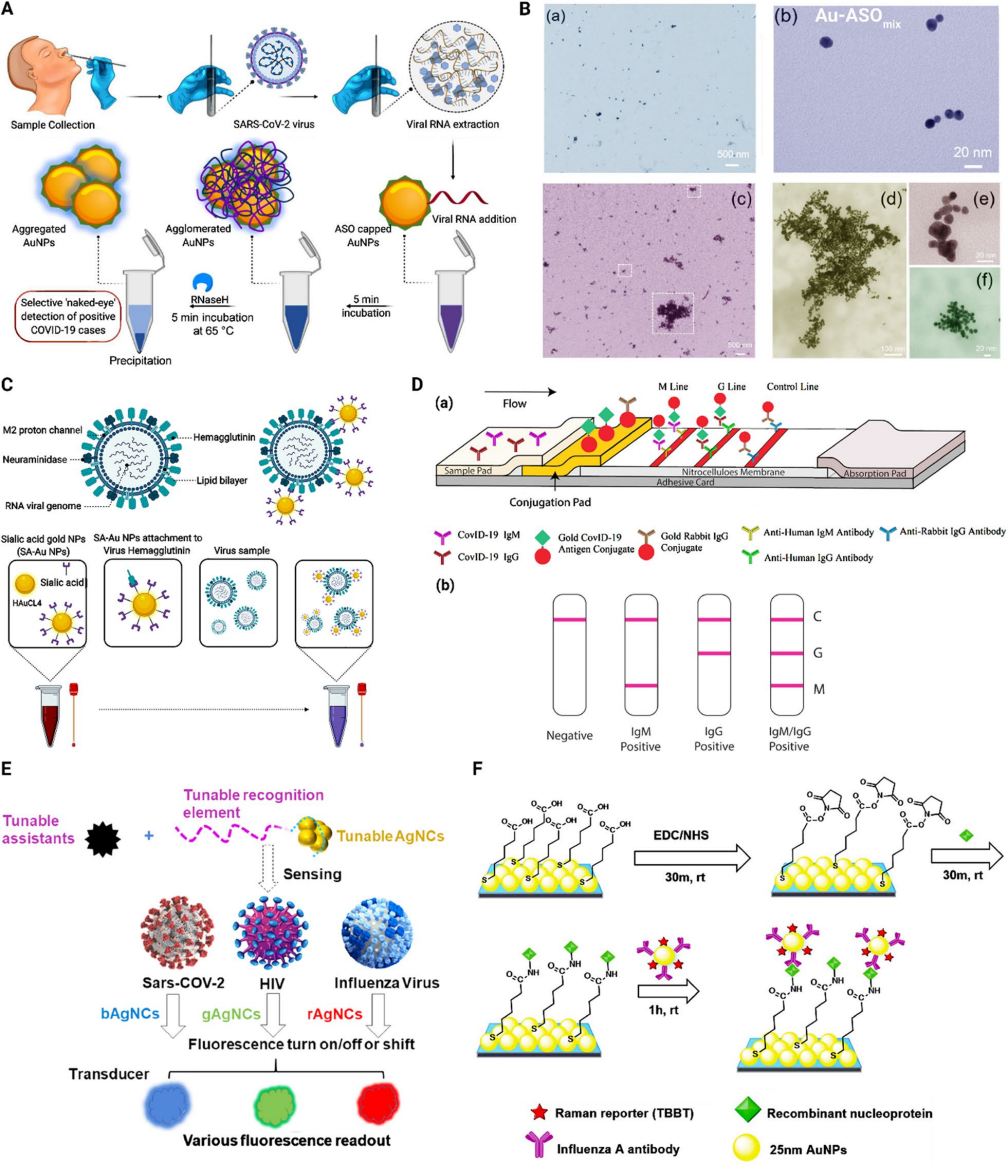
Examples of metallic nanoparticle-based strategies for diagnosis of respiratory viruses.** A** A schematic of the ‘naked-eye’ diagnosis of SARS-CoV-2 through proper design of ASO-capped gold nanoparticles (AuNPs). Reprinted from [148] with permission from the American Chemical Society. **B**—**a** Transmission electron microscopy (TEM) of ASO-functionalized AuNPs, (b) TEM image of the individual ASO-functionalized AuNPs, **c**–**f** TEM images of ASO functionalized AuNPs after addition of SARS-CoV-2 RNA. Reproduced from [148] with permission from the American Chemical Society. **C** Schematic of the preparation of a diagnostic system for SARS-CoV-2, influenza B and MERS-CoV viruses Reprinted from [149]. **D**—**a** Schematic of a point-of‐care lateral flow immunoassay for rapid detection of SARS‐CoV‐2 IgM‐IgG antibodies. **b** Schematic on the test results. C, control line; G, IgG line; M, IgM line. Reproduced from [155] with permission from Wiley. **E** Schematic of DNA-AgNCs platform for virus detection through fluorescent readouts based on fluorescence enhancement (turn on), quenching (turn off), or color shifts. Reprinted from [157] with permission from the American Chemical Society. **F** Schematic of a two-dimensional array of Au@Ag core-shell nanoparticles for direct immunoassay of influenza A virus. Reprinted from [161] with permission from the Royal Society of Chemistry. *IgG* immunoglobulin G, *IgM* immunoglobulin M, *AgNCs* silver nanoclusters, *SARS‐CoV‐2* severe acute respiratory syndrome coronavirus 2, *EDC* 1-ethyl-3-(3-dimethylaminopropyl) carbodiimide, *NHS* *N*-hydroxysuccinimide, *rt* room temperature, *MHDA* 16-mercaptohexadecanoic acid

Gold nanoparticles were the first nanomaterials developed for virus detection. They were investigated in the late 1990s for human papillomavirus detection [145]. Metallic nanoparticles, especially gold nanoparticles, exhibit promising properties for nano assays because of their easy manufacturing, characterization, and surface modification, stability, good biocompatibility, and high adsorption constant. In addition, they are chemically stable and possess water solubility as well as size and shape controllability; along with their tunable photochemical/physical characteristics, which makes them suitable nano-probes for optical (bio)sensors. They are available in different shapes, including spheres, rods, prisms, tetrapods, cubes, shells, and hollow structures, and interestingly, different morphologies lead to different excitation/emission ranges [8]. These nanoparticles are helpful for sensitive, specific sensing and detection because they readily form active and strong bio-conjugates with targeting biomolecules such as proteins and DNA. They have been extensively employed for virus detection because their surface may be electrostatically decorated with moieties like antigens and antibodies. Gold nanoparticles may be used as probes via plasmon resonance shift, surface-enhanced Raman spectroscopy (SERS), as well as naked-eye monitoring via color-changing [8, 146]. They possess high free-electron surface density, known as “plasmons”. Surface plasmon bands are very sensitive to nanoparticles’ shape and interparticle distance, though it is less dependent on the size of the nanoparticles. For instance, surface plasmon resonance of gold nanoparticles can cause a significant enhancing or quenching effect due to interactions with adjacent photon emitters [147]. A colorimetric assay employing gold nanoparticles functionalized with thiol-modified antisense oligonucleotides (ASOs), particularly for the N-gene (nucleocapsid phosphoprotein) of SARS-CoV-2, was conducted. The thiol-modified ASO functionalized gold nanoparticles selectively agglomerate after they came in contact with their specifically targeted RNA sequence. The agglomerated nanoparticles induced alteration in their surface plasmon resonance. This system is applicable for diagnosis of SARS-COV-2 within 10 min and has been tested in the presence of RNA from MERS-CoV, resulting in a detection limit of 0.18 ng/µL of SARS-CoV-2 viral RNA (Fig. 7A, B) [148]. Gold nanoparticles conjugated to sialic acid, a glycoprotein available on the surface of lung epithelial cells, were investigated for their potential in diagnosing respiratory viruses, mainly SARS-CoV-2, as well as Influenza B and MERS-CoV viruses. This system changed color through its plasmonic shift as soon as the conjugated nanoparticles were bound to viruses (Fig. 7C) [149].

Silver nanoparticles have a broad range of biomedical applications because of their selective toxicity against microorganisms. These nanoparticles have a very reactive surface area as well as superior optical and catalytic properties despite their colloidal nature. A decrease in the size of silver nanoparticles results in increases in their specific surface area. This, in turn, causes considerable changes in biological, physical, and chemical activities [150, 151].

Surface-enhanced Raman spectroscopy (SERS) possesses significantly magnified vibrational signatures of extremely low-concentration molecules. Incident laser intensity and surface plasmon resonance coupling in nanostructured metal surfaces is the main origin for huge field enhancement, leading to the Raman cross-section of the analyte [152, 153]. The spike proteins of CoVs are considered crucial structural proteins for viral entry and disease pathogenesis. Gold nanoparticles with the attachment of anti-spike antibodies have been used for rapid screening of patients with SARS-CoV-2 infection within 5 min. For this purpose, 4-amino thiophenol was attached to the gold nanoparticles with an Au-S bond and developed for SERS. Upon contact with SARS-COV-2 antigen or virus particles, the antigen-antibody reaction causes aggregation of the gold nanoparticles, resulting in a color change from pink to blue that enables virus detection by the naked eye [22]. It should be noted that, by changing the binding/interacted atoms to the thiol functional groups of the virus, their interaction distances change, therefore it caused different optochemical/physical characteristics and finally different limits of detection. Therefore, investigating the chemical structure of different small molecules-decorated on the surface of the nanoparticles that interacted with the proteins of SARS-CoV-2 would be of great importance.

Point-of-care testing is designed for medical testing at or near the place where patients first face the health care system. Rapid strips are extensively employed for many point-of-care testing applications owing to their superior characteristics such as rapid diagnosis, easy operation, and inexpensiveness [154]. A point-of-care detection kit was designed based on lateral flow immunoassay. Gold nanoparticles were employed for the simultaneous detection of IgM and IgG antibodies against SARS-CoV-2 in human blood. Results may be obtained within 15 min, with 88.7% sensitivity and 90.6% specificity (Fig. 7D) [155]. A rapid SARS-CoV-2 detection method based on gold nanoparticle lateral flow strips was developed utilizing colloidal gold nanoparticles. The latter is easy to synthesize and possesses long-term stability, naked-eye detectability, as well as biocompatibility. In this method, IgM conjugated to AuNPs was used for virus detection via indirect immunochromatography. Results may be obtained within 15 min with 10–20 µL of serum for each test [156].

Metallic nanoclusters are a category of nanomaterials that contain a small number of atoms of a precise number. Although metallic nanoparticles exhibit a surface plasmon effect, metallic nanoclusters possess fluorescence with remarkable photostability. Metallic nanoclusters are manufactured by metal cores (mostly Au, Ag, Cu; and rarely other types of optical/electrochemical active transition metals including Pd, Pt, and Zr) and stabilized with ligands or templates. The latter are small molecules instead of large molecules such as DNA, peptide, and polymers. Silver nanoclusters have gained attention among metallic nanoclusters due to their small size, high photostability, non-toxicity, and biocompatibility. Silver nanoclusters tend to exhibit brighter fluorescence in comparison to gold nanoclusters and copper nanoclusters with identical stabilizing ligands. Additionally, silver nanoclusters stabilized with DNA produce ultra-bright fluorescence (comparable with highly fluorescent fluorophores), have easy synthesis, and tunable fluorescence properties (Fig. 7E) [157, 158]. DNA hairpin structure consisting of guanine-rich sequences (GRSs) at the two terminals can enhance the fluorescence of silver nanoclusters. This combination was designed to produce a probe for detecting viral disease-related genes. The system is a highly sensitive and selective method for DNA analysis with the capability to distinguish as minute as one mismatched nucleotide target [159]. The systems based on silver nanoparticles can exhibit unique and pre-defined molecular interactions with the SARS-CoV-2, due to their tunable size and also their ability to conjugate with a wide range of natural components. Also, different homogenous and heterogenous surface decorations would be a wise choice for multi-targetings approaches.

Core-shell nanoparticles are superior to ordinary nanoparticles as they have lower cytotoxicity, higher dispersibility, better biocompatibility, and cytocompatibility, as well as enhanced thermo-chemical stability [160]. For instance, two-dimensional arrays of Au@Ag core-shell nanoparticles have been investigated as a SERS substrate for improving the sensitivity of influenza A virus detection. This system significantly improved the SERS signal compared to a flat Au film (Fig. 7F) [161].

Reverse transcription polymerase chain reaction (RT-PCR) methods have been extensively used for SARS-CoV-2 diagnosis. Efficient RT-PCR assays require quantitative and qualitative extraction of highly-pure nucleic acid. Conventional RNA extraction methods require numerous centrifuging and column-transferring stages that hinder the use of RT-PCR for rapid diagnosis. Apart from being time-consuming laboratory processes, they are vulnerable to contamination and column clogging [162]. This problem has been solved with the use of magnetic nanoparticles. The magnetic nanoparticles consist of a diversity of materials such as iron oxide, cobalt oxide, and nickel oxide within a size range between 1 and 100 nm. They possess superior properties such as high specific surface area and favorable magnetic characteristics that are size-dependent and different from bulk materials. Magnetic nanoparticles have gained remarkable attention in diagnostic medicine because of their special optics after suitable functionalization, magnetism, electricity, chemical, mechanical and thermal properties [163, 164]. Magnetic nanoparticle-based extraction assays are simple to operate, centrifuge-free, and compliant with automation. Despite being easier and faster compared to conventional methods, common magnetic nanoparticle-based extraction methods still require processing stages such as lysis, binding, washing, and elution. These procedures create functional complexity for clinical diagnosis [165]. Magnetic nanoparticles functionalized with poly(amino ester) and carboxyl groups have been developed as an easy and efficient RNA extraction method for sensitive diagnosis of SARS-CoV-2 RNA by RT-PCR. This method combined virus lysis and RNA binding stages to simplify the extraction method. The system has the potential to be adopted for entirely automated nucleic acid extraction systems [165].

Iron (II, III) oxide (Fe_3_O_4_) magnetic nanobeads have been used for designing a test strip for SARS-CoV-2 diagnosis based on a double antigen sandwich. Briefly, Fe_3_O_4_ magnetic nanobeads were coupled to specific antibodies. The S protein of SARS-CoV-2 was used as a coating antigen to capture specific antibodies against SARS-CoV-2. This method may be used for the clinical diagnosis of samples containing specific antibodies directed against viral nucleoprotein. The unique characteristics of Fe_3_O_4_ magnetic nanobeads make it possible for qualitative and quantitative detection of SARS-CoV-2 antibodies (IgG and IgM) in serum via immunochromatography. The designed kit has the potential to detect samples within 15 min, with high sensitivity [166]. Moreover, a magnetic immunoassay was designed for the detection of influenza A virus subtype H1N1. The immunoassay utilized magnetic particle spectroscopy and the self-assembly of magnetic nanoparticles to quantitatively diagnose H1N1 nucleoprotein molecules. Magnetic nanoparticles were functionalized with IgG antibodies and cross-linked to influenza A (H1N1) nucleoproteins to create magnetic nanoparticle clusters. Magnetic particle spectroscopy was used to track the harmonics of the oscillating magnetic nanoparticles as an indicator of rotational process freedom, representing the bound states of the magnetic nanoparticles. These harmonics could be rapidly and easily collected from nanogram quantities of Fe_3_O_4_ nanoparticles in 10 s. The experimental assay may be used as a fast, sensitive, and wash-free magnetic immunoassay [167]. Due to the importance of the preparation cost of the final products, those iron oxide nanoparticles can be able to synthesized via green methods, and decorated with natural-derived components from leaf extracts. Also, those natural components and leaves contain several functional groups that make them suitable for making core–shell nanostructures.

Copper nanoparticles have good biocompatibility, low toxicity, and oxidation resistance. They are readily available, inexpensive, and possess antiviral, antifungal, and antibacterial activities. Despite their promising characteristics, copper nanoparticles have not been studied as much as other metallic nanoparticles such as gold nanoparticles and silver nanoparticles [168, 169]. Metallic nanoparticles derived from copper, silver, and gold were experimentally used for the diagnosis of RSV. RSV is a paramyxovirus and the most common reason for childhood acute respiratory infection, It is one of the leading causes of hospitalization in infancy [170]. The aforementioned nanoparticles were functionalized with anti-RSV antibodies and used for localized surface plasmon resonance shifting as a method for RSV detection. The specificity of the functionalized nanoparticles for RSV detection was evaluated in the concomitant presence of adenovirus and the bacterium *Pseudomonas aeruginosa*. The functionalized copper and silver nanoparticles were specific for RSV detection but did not demonstrate any plasmon resonance shift in the presence of *P. aeruginosa* and the adenovirus. The functionalized copper nanoparticles were preferred for RSV detection over the silver and gold nanoparticles based on the detection and quantification values [171].

Silica nanoparticles have broad applications in biomedical science because of their biocompatibility, large specific surface area, pore-volume, adjustable particle size, as well as easy and low-cost synthesis. Biomolecules such as peptides, DNA, antigens, and antibodies can be linked to silica nanoparticles. Hence, silicon is a promising element to be incorporated in biosensors and immunosensors [137, 172]. Silica nanoparticles were used for SARS-COV-2 diagnosis after coating with two redox dyes (methylene blue and acridine orange), yielding silica-methylene blue and silica-acridine orange. The dye-coated silica nanoparticles were used for electrochemical measurements through a potentiostat instrument. Using this method, both the nucleocapsid and the spike protein have a limit of detection of 1 copy/µL. More importantly, the equipment used for measurement (potentiostat) is transportable, which enables on-site testing for SARS-COV-2. This platform is a one-step, rapid (2 h) sandwich hybridization assay that has high specificity, sensitivity, and accuracy [173].

A novel silicon nanowire-based sensor was designed and functionalized with an anti-COVID-19 spike protein antibody for the recognition of COVID-19 viral particles via a semiempirical modeling method. It was reported that the silicon nanowire-based sensor has high sensitivity and selectivity for accurate COVID-19 detection than other viruses e.g., influenza, rotavirus, and HIV [174].

**Fig. 8:**
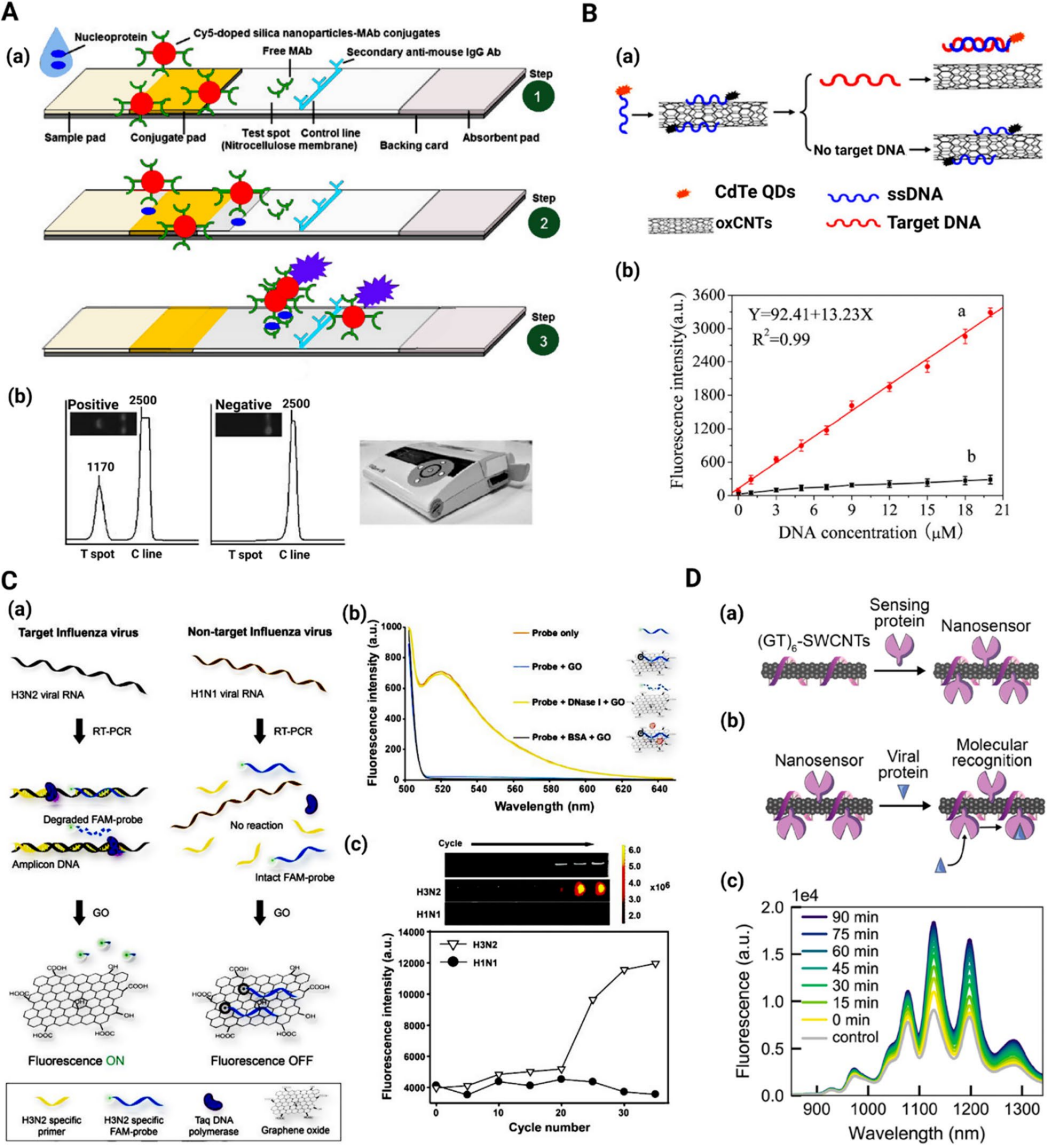
Non-metallic nanoparticle-based strategies for diagnosis of respiratory viruses. **A** Schematic of **a** the fluorescence-based lateral flow immunoassay; step 1 depicts the system principle, step 2 shows antigen binding to the conjugates and their flow along the strip to the test spot, and step 3 displays capturing of flow excess conjugates. Reproduced from [175] with permission from Springer. **B**—**a** Schematic of fluorescence resonance energy transfer (FRET)-based system for H5N1 influenza detection; **b** plots of fluorescence intensity exhibited by different concentrations of target H5N1 ssDNA (red graph) and non-target DNA (black graph). Reproduced from [183] with permission from Elsevier. **C**—**a** Schematic of the fluorometric system for detection of influenza subtypes viral genes; **b** DNase I treatment and fluorescence difference after GO incubation; **c** increase in fluorescein amidite (FAM)-DNA probe fluorescence with PCR cycle progression Reproduced from [188] with permission from Elsevier. **D**—**a** Schematic of ACE2-SWCNT nanosensor formation; **b** Schematic of ACE2-SWCNT nanosensor interacting with viral spike protein; **c** fluorescence spectrum showing strong turn-on fluorescence response after addition of 10 mg/L SARS-CoV-2 spike protein (final concentration) to the ACE2-SWCNTs. Reproduced from [191] with permission from the American Chemical Society

New diagnostic strategies based on the use of fluorescent nanomaterials are rapidly gaining impetus. For instance, fluorescent silica nanoparticles have been employed for fabricating influenza A antigen detection test strips based on lateral flow immunoassay. This method benefits from the high brightness and photostability of the Cy5 dye-doped silica nanoparticles, as well as the rapidity of the lateral flow immunoassay. The nucleoprotein of the influenza A virus could be detected as low as 250 ng mL^−1^ using a 100 µL sample, within 30 min, without interference from other proteins (Fig. 8A) [175].

Self-fluorescent nanoparticles have been used for the development of fluorescent probes. Among these, carbon dots are the best-investigated carbon-based fluorescent nanoparticles [176, 177]. Because of their excellent optical, luminosity, physical, and chemical properties, carbon dots and quantum dots (such as CdS, CdSe, and PbSe) are expected to replace conventional phosphor materials in the near future [178]. Conventional metallic quantum dots suffer from limitations such as toxicity, hydrophobicity, high cost, and complex synthesis process [179, 180]. As a new member of the fluorescent carbon material with a diameter below 10 nm, carbon dots are a promising alternative to metal-based quantum dots for the preparation of fluorescent probes because of their composition and biocompatibility [181, 182]. Carbon dots may be synthesized from diverse chemically or green sources such as citric acid, sucrose, and glucose [178]. In one study, the detection of H5N1 influenza virus was achieved via fluorescence resonance energy transfer (FRET) from CdTe quantum dots to carbon nanotubes. In this approach, quantum dots modified with ssDNA were used as donors. The quantum dots were effectually quenched in the primary stage by the strong interaction between ssDNA and the carbon nanotubes. After target recognition, competitive binding to the quantum dots-ssDNA resulted in the removal of oxidized carbon nanotubes and quantum dots fluorescence recovery. The recovered fluorescence of the quantum dots was linearly proportional to the target concentration (0.01–20 µM), with a limit of detection of 9.39 nM (Fig. 8B) [183].

Graphene is a carbon-based material that is obtained by graphite mechanical exfoliation. Graphene is a hydrophobic compound with layers adhering together in stacked mode on top of each other via π–π-stacking interactions [184, 185]. Graphene oxide is a soluble, two-dimensional (2D) atomic crystal containing sp^2^-hybridized carbon atoms consisting of epoxy, hydroxyl, and carboxyl functional groups. Apart from possessing superior electronic, thermal, and mechanical characteristics as well as good chemical stability, graphene oxide is biocompatible, nontoxic, non-expensive, possesses a high specific surface area, and is soluble in water. Graphene oxide demonstrates an excellent capability to provide a chemically adjustable platform for conjugation due to the strong noncovalent interactions of their 2D surface with adsorbed biomolecules through the π–π stack electrostatic forces or hydrogen bonding [186, 187]. Graphene oxide has been used to design a fluorometric system for the detection of influenza subtypes viral genes. A fluorescent DNA probe related to the influenza virus hemagglutinin gene became hydrolyzed due to the 5′ to 3′ exonuclease activity of *Taq* polymerase during the PCR. After incubation with graphene oxide, the emitted fluorophore was not adsorbed on the graphene oxide and retained its fluorescence. In the absence of target influenza chiral RNA, the unchanged fluorescent DNA probe readily adsorbed graphene oxide with quenched fluorescence. The multi-well plate system has the potential to detect 3.8 pg of influenza viral RNA (Fig. 8C) [188].

Carbon nanotubes are graphite sheets that are rolled up into tube-like shapes with nanometer-scale diameters. Based on the number of carbon layers, they may be divided into single-walled carbon nanotubes (SWCNTs) and multi-walled carbon nanotubes (MWCNTs). Carbon nanotubes display specific structural, functional, thermal, electrical, chemical, and optical properties, which make them valuable for biomedical applications such as the fabrication of biosensors [189, 190]. For instance, a SARS-CoV-2 detection system was developed using SWCNTs functionalized with ACE2. Identification of S RBD by the nanosensor yielded a strong turn-on fluorescence response. The SWCNT-based nanosensors have the potential to detect SARS-CoV-2 S protein and can be immobilized and imaged on microfluidic surfaces (Fig. 8D) [191]. The mechanism of these MWCNT-based compartments is based on the host–guest interactions with the biomarkers/virus, which is completely a physical interaction.

## Nanostructures for the treatment of viral respiratory infections

Despite significant efforts to develop an effective treatment against different types of respiratory viruses, there is still no precise strategy with highly predictable results. The multi-drug resistance of various viral strains, the spreading of viruses into inaccessible anatomical regions (e.g., lymphatic system, CNS, and synovial fluid), as well as the capability of viruses to remain in their latent stage for a prolonged time, are major challenges that have to be addressed for successful clinical use of antiviral therapy [192, 193]. Nanotechnology can be used to overcome the challenges associated with antiviral therapy and to generate new therapeutic strategies for eradicating viral disorders. Nanomaterials are appropriate candidates for the treatment of viral infections because of their excellent physicochemical characteristics, including high surface area, nano-size dimensions, and surface modification potential [194, 195]. To date, numerous nano-based strategies have been developed to face viral respiratory diseases. These strategies are summarized in this section.

### Nanoparticles as antiviral therapeutics

Nanoparticle-based therapeutics can inhibit viral infection in several ways, including blocking the binding of viruses to their host receptors, preventing viral replication, as well as directly inactivating the viruses [196]. For example, the boronic acid side chain of carbon-quantum dots interacts with the S glycoprotein receptors of human coronavirus 229E (HCoV-229E), restricting the bond between the virus and host cell receptors, and also affecting the genomic replication of the virus [197].

Metal nanoparticles are effective in immunomodulation acting as inflammation inhibitors, and possess antiviral effects. Gold nanoparticles stimulate macrophage functions and promote phagocytosis to improve immune responses. Gold, silver, and copper nanoparticles inhibit the cytokine storm after SARS-CoV-2 infection. Gold nanoparticles have been demonstrated to reduce cytokine production and increase both cellular and humoral immune responses. Similarly, copper nanoparticles inhibit inflammatory activity by the reduction of interleukin and TNF-α release. The antiviral effects of gold nanoparticles are also attributed to the disruption of the outer layer of the coronavirus, whereas the antiviral effect of copper nanoparticles is attributed to the degradation of the viral capsid [198].

Silver nanoparticles exhibit similar antiviral and immunomodulatory proprieties against respiratory pathogens such as adenovirus, RSV, and influenza [199–201]. Silver nanoparticles in the range of 1–10 nm have a strong binding affinity to viruses. This prevents the virus from binding to host cells [202]. Silver nanoparticles have a high affinity toward the sulfur present in amino acids such as cysteine and glutathione, as well as the sulfhydryl groups present in the active sites of different enzymes [80]. Silver nanoparticles disrupted the mitochondrial network and prevented the translocation of the antiviral IRF-7 transcription factor into the nucleus of the lung cells. By blocking the autophagic flux, viral replication and activation of the pro-inflammatory responses mediated by neutrophils were inhibited [203]. Zero-valent silver nanoparticles (Ag^0^) released Ag^+^ ions and generated ROS to damage viral DNA [204, 205].

Curcumin is an efficient antiviral agent. It was immobilized on the surface of silver nanoparticles to enhance their inhibitory effects against the entry of respiratory viruses into the host cells [206]. Smaller curcumin-immobilized silver nanoparticles had larger surface areas and were less cytotoxic and more effective than larger nanoparticles. These events are related to “protein corona” formation, with the larger surface areas of small nanoparticles having more direct interactions with the viral proteins to generate a better inhibition effect. Silver nanoclusters also possess good antiviral activity against some viruses. A recent study reported the use of silver nanoclusters in combination with glutathione to target the porcine epidemic diarrhea virus [205]. The synthesis of negative-strand RNA and viral budding was hampered once the nanoclusters encountered the viruses. These nanoclusters may be used as a therapeutic drug for many respiratory pathogens such as SARS-CoV-2.

**Fig. 9:**
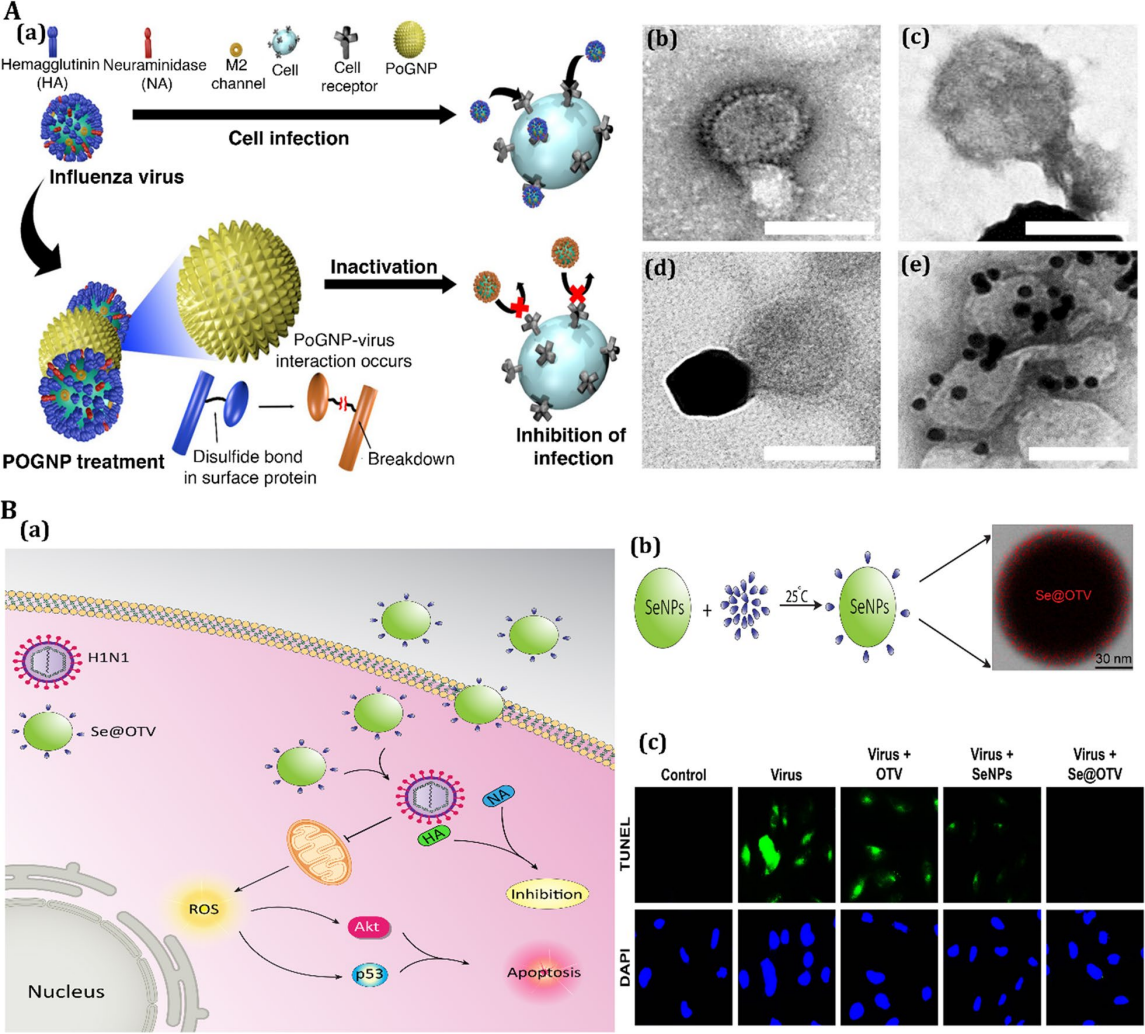
Antiviral activity of nanomaterials for treatment of respiratory viral infection.** A** Antiviral mechanism of porous gold nanoparticle (PoGNP) against Influenza A virus (IAV): **a** PoGNP interacts with IAV surface proteins and cleaves their disulfide bonds. Inactivated viruses exhibit lower infectivity to cells, **b** TEM image of IAV, **c** PoGNP-treated IAV, **c** spherical gold nanoparticle-treated IAV, **d** AgNP-treated IAV. Reprinted from [210] with permission from Springer. **B** Antiviral mechanism of selenium nanoparticles (SeNPs) modified with oseltamivir (OTV) called as Se@OTV against H1N1: **a** Se@OTV-induced apoptosis in H1N1 infection of MDCK cells, **b** synthesis rout of Se@OTV, **c** intracellular apoptotic signaling pathways by Se@OTV in H1N1 infection of MDCK (Madin–Darby canine kidney) cells. All panel except **B** (**a**) reprinted from [214] with permission from Dovepress

Gold nanoparticles have been investigated extensively for clinical antiviral applications because of their low cytotoxicity compared to other metal nanoparticles [207]. They activate the immune system and stimulate adaptive immune responses by direct interference with the cell entry mechanism [208]. Modification of Au nanorods with a “pregnancy-induced hypertension” peptide blocked the formation of MERS-CoV heptad repeat 1 domain (HR1)/HR2 complex, inhibiting the cell fusion process with higher efficiency than the peptide alone [209]. In a study, porous gold NPs were developed for cleaving disulfide bonds in order to improve antiviral treatment agents (Fig. 9A) [210]. The porous gold NPs allow for a simple production technique that does not require any extra heat or surfactant, whereas Ag-based NPs should be modified with a capping agent for in vitro stability and to minimize their cytotoxicity. Besides, porous gold NPs also have a large surface area, thanks to their unique nanobundled structure. As a result of the formation of gold-disulfide bonds, porous gold NPs are predicted to have a high affinity for disulfide bonds. Also, their large surface area can effectively inactivate the influenza virus by cleaving disulfide links, preventing membrane fusion and viral entrance into the host cell [210].

Porous silica nanoparticles possess low host cell cytotoxicity and are capable of blocking viral entry. These nanoparticles bind to viral membranes (such as HIV-1 or RSV) and act as virus scavengers to prevent them from attaching to the host cells [211]. Similarly, mesoporous silica nanoparticles bind to enveloped viruses via hydrophobic/hydrophilic interactions. The strong bonds between silica and the viral membrane can break the attachment of viruses to the host cell and block viral internalization [212]. Selenium nanoparticles are biocompatible and have gained attention because of their antiviral effects. The antiviral activity of selenium nanoparticles may be enhanced when they are integrated with antiviral therapeutics. For instance, the use of selenium nanoparticles with the antiviral drug arbidol blocked cell entry of the H1N1 influenza virus and minimized virus-mediated cell apoptosis [213]. In another work, oseltamivir-adorned selenium NPs with remarkable antiviral activity and restriction on drug resistance were developed (Fig. 9B) [214]. Oseltamivir-decorated SeNPs suppressed H1N1 infection and were less cytotoxic than SeNPs. Oseltamivir-decorated SeNPs inhibited the action of hemagglutinin and neuraminidase, preventing the H1N1 influenza virus from infecting host cells. Oseltamivir-adorned selenium NPs were able to prevent H1N1 from infecting Madin-Darby canine kidney (MDCK) cells along with avoiding chromatin condensation and DNA fragmentation. Furthermore, oseltamivir-decorated SeNPs disallowed the production of ROS as well as the phosphorylation of p53 and the activation of Akt. These findings show that oseltamivir-decorated SeNPs are a potential and effective antiviral drug for H1N1 [214].

Iron nanoparticles are also effective against respiratory viruses such as multiple pandemic influenza viruses [215]. A molecular docking study of iron oxide nanoparticles demonstrated efficient interaction of Fe_2_O_3_ and Fe_3_O_4_ nanoparticles with SARS-CoV-2 S RBD, inducing spike conformational changes that led to virus inactivation [216]. Zirconia nanoparticles inhibited the replication of the H5N1 influenza virus in mice lung epithelial cells [217]. The antiviral mechanism of zirconia nanoparticles involves the induction of innate immunity and the production of pro-inflammatory cytokines associated with antiviral immune responses. This strategy protected lung epithelial cells against influenza infection and sheds light on the potential use of zirconia nanoparticles in treating other virus-induced respiratory disorders.

Graphene-derived nanoparticles such as graphene oxide and reduced graphene oxide also have similar inactivation effects against viruses [218]. Their antiviral property is attributed to their negative charges and the sharp edges of the graphene sheets, which are effective in interfering with the binding of the viruses to host cells [219, 220]. The surface of graphene oxide may be modified with antiviral agents to exert a synergistic effect against viruses. For example, graphene oxide sheets may be used as a stabilizing agent to inhibit silver nanoparticle agglomeration and reduce cell toxicity [221]. The graphene oxide-silver nanoparticles demonstrated augmented antiviral effects toward enveloped viruses. Graphene oxide surfaces may also be functionalized with β-cyclodextrin and curcumin for use against RSV [222]. This platform prevented RSV from infecting the host cells through the inhibition of virus attachment to the host cell. In addition, modification of the graphene oxide platform with sulfate groups increased the affinity of the graphene oxide toward viruses because the sulfate groups enabled multivalent interactions between the graphene oxide and viruses [223].

A hollow polymeric nanoparticle was prepared from poly (lactic-co-glycolic acid) (PLGA) with core–shell morphology to progress an antiviral drug and vaccine against MERS-Cov. It was reported that the hollow polymeric nanoparticle-based vaccine prevented viral infection, was immunogenic, and hindered the introduction of unfavorable lung illness in immunized human DPP4 enzyme transgenic mice [224].

Finally, polymer-based nanoparticles such as chitosan also possess antiviral activity, being able to potentially interact with the S proteins of coronaviruses and inhibit their attachment to the ACE2 receptor [225].

### Nanoparticle-assisted gene therapy against viruses

Nanoscale carriers can be employed to augment the transfection efficacy of small interfering RNA (siRNA) and clustered regularly interspaced palindromic repeat (CRISPR)/CRISPR-associated (Cas) (CRISPR/Cas) systems into target cells [226, 227]. These systems are effective approaches to interfere with and block the replication of RNA viruses. Antiviral siRNA and CRISPR therapy possess unique advantages in comparison to conventional antiviral drugs. These advantages include high sensitivity and selectivity toward the targeted sequence, rapid action at different viral stages, and the need for less amount of siRNA to downregulate viral RNA [228].

The RNA interference technique was successfully used to combat human respiratory viruses. Different respiratory viruses, including RSV, influenza [229], SARS-CoV [230], MERS-CoV [231], and SARS-CoV-2 [232], have been used as targets for experimental siRNA-based therapy aimed to reduce viral titers. Unlike siRNA, CRISPR/Cas is a gene-editing technique based on a form of immunity developed by bacteria, which allows DNA cleavage in specific regions [233]. The CRISPR/Cas systems have been applied to obtain specific gene knockouts, to direct precise DNA insertion, chromosomal rearrangement, or to regulate gene expression to establish cell and animal models of respiratory disorders or for anti-viral therapy against respiratory viruses [234]. Likewise, CRISPR provides high-throughput screening for antiviral drug discovery through CRISPR guide RNA (gRNA) libraries, with promising applications against viral respiratory infections [235]. CRISPR/Cas9 and siRNA delivery are obtained through plasmid or mRNA transfection or by viral vectors. Nevertheless, this latter approach has some drawbacks [236]. For example, viral vectors have limited carrying capacity. In addition, the viral vectors are cleared quickly from systemic circulation. They also carry a high carcinogenesis potential and can elicit immune responses, which decrease the chance of successful siRNA/CRISPR delivery [226, 227].

**Fig. 10:**
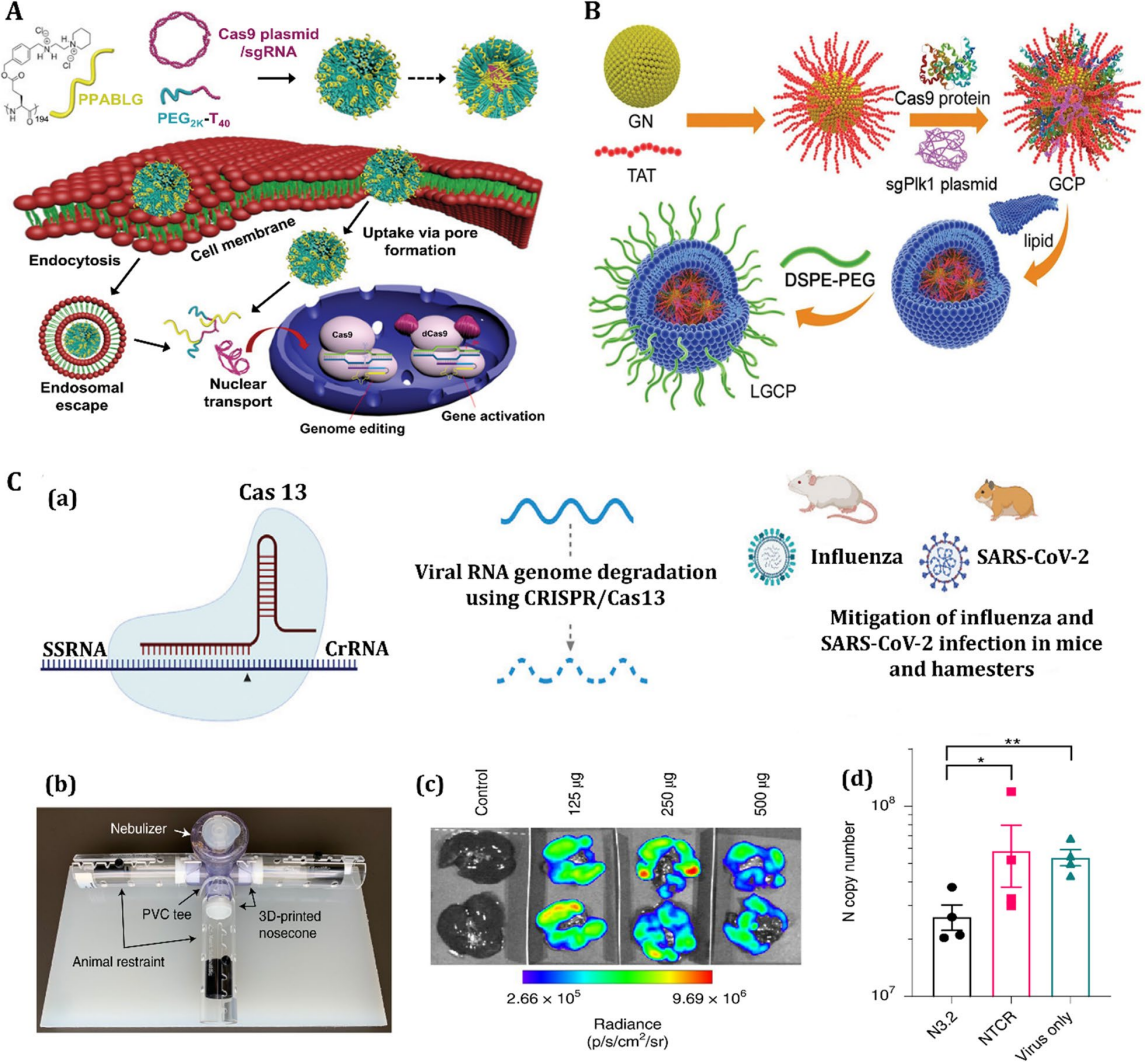
An overview of the different applications of clustered regularly interspaced palindromic repeat (CRISPR)/CRISPR-associated (Cas) system for in vivo genome editing. **A** Schematic representation of PEGylated nanoparticles based on the α-helical polypeptide PPABLG (P-HNPs) and their intracellular transport of Cas9 expression plasmid/single guide RNA for genome editing or gene activation. Reprinted from [246] with permission from National Academy of Sciences. **B** Schematic representation of the LGCP (polyethylene glycol-lipid/gold nanoclusters/Cas9 protein/sgPlk1 plasmid) fabrication process. LGCP delivering Cas9 protein/sgPlk1 plasmid successfully inhibited the progression of the disease by knocking-out the Plk1 gene. Reprinted from [247] with permission from John Wiley & Sons, Inc. **C** Schematic representation of CRISPR/Cas systems as antiviral therapy: **a** Cas13 cuts ssRNA and could be used to degrade viral RNA genomes, **b** inhalable antiviral Cas13a mRNA in rodents, **c** Hamsters were dosed as indicated with aNLuc mRNA. Lungs were analyzed at 1 d for luminescence, and **d** lung viral loads from hamsters at 6 d after infection (n = 4). Data represent mean N copy number ± s.e.m. Brown–Forsythe and Welch ANOVA with Dunnett’s multiple comparisons on log-transformed data, where **P = 0.0016 and *P = 0.0198. Reprinted from [248] with permission from Nature

Biocompatible and non-toxic nanocarriers can be efficacious tools for in vivo siRNA/CRISPR delivery. The nanocarriers protect the nucleic acids from degradation by serum nucleases. This helps to improve their stability and prolong their half-lives. When functionalized nanoparticles are used for localized delivery of siRNA, the systemic toxicity of the siRNA cargo is also reduced. A plethora of nanocarriers has been developed for the improved delivery and administration of siRNA/CRISPR. These nanocarriers include lipoplexes, polyplexes, lipid nanoparticles, gold nanoparticles, iron oxide nanoparticles, nanohydrogels, silica, and cell-penetrating peptides [237–239]. These nanocarriers do not trigger a strong immune reaction, are easy to be scaled up in their manufacturing, and increase the uptake of siRNA/CRISPR to reduce off-target effects [240–242]. The lipid and polymer-based nanocarriers have been approved by the United States Food and Drug Administration (FDA) to be used as functional platforms for siRNA/CRISPR delivery because of their positive charges and their highly biocompatible and biodegradable attributes [243–245]. Currently, multiple synthetic vectors have been established to successfully deliver CRISPR/Cas9 systems designed to knock out disorder-related genes and reduce the progression of the disease. For instance, PEGylated NPs based on the α-helical polypeptide PPABLG (P-HNPs) were fabricated to co-deliver Cas9 expression plasmids and sgRNAs targeting polo-like kinase 1 (Plk1), called P-HNPPCas9 + sgPlk1 [246]. The gene editing efficiency at the Plk1 locus was found to be 35%, which resulted in significant knockout gene suppression (more than 71%) and improved the survival rate of injected mice (60%) (Fig. 10A). To absorb Cas9protein/sgPlk1 plasmid, TAT-peptide (GRKKRRQRRRPQ) coated gold nanoclusters were employed as a core. The nanoplatform was encased in a cationic lipid shell after absorption to generate a lipid-coated nanosystem [247]. The injection of lipid-coated nanosystem successfully reduced the progression of the disease by eliminating the Plk1 gene in the desired site and drastically down-regulating Plk1 protein expression (Fig. 10B). More recently, Blanchard et al. also offered CRISPR/Cas systems as antiviral therapy against Influenza and SARS-CoV-2 using mRNA-encoded Cas13a (Fig. 10C) [248]. They developed CRISPR RNAs (crRNAs) specific for influenza virus PB1 and highly conserved portions of PB2, as well as the replicas and nucleocapsid genes of SARS-CoV-2, and identified the crRNAs that suppressed viral RNA levels in cell culture most effectively. They used a nebulizer for administering polymer-formulated Cas13a mRNA and verifying guides to the respiratory system. Cas13a efficiently eliminated influenza RNA in lung tissue in mice following infection, whereas Cas13a delivery reduced SARS-CoV-2 replication and symptoms in hamsters. The findings show that Cas13a-mediated virus targeting can help to prevent respiratory infections [248].

Commercially available cationic lipid carriers such as lipofectamine (Invitrogen, Waltham, MA, USA), lipofectin, RNAifect (Qiagen, Hilden, Germany), oligofectamine, TransIT-TKO (Mirus, Madison, WI, USA) have shown reliable results for the delivery of antiviral siRNA [249]. These polycationic lipids preserve the low endosomal pH by enhancing the influx of protons and water, resulting in enhanced release of the loaded therapeutics from the endosome into the cytosol of the lung cells [249]. Poly(lactic-co-glycolic acid), lipid, and polymer-lipid nanocarriers are also promising candidates for the delivery of aerosol-based pulmonary antiviral siRNA and inhalable antiviral siRNA [250]. Nanoparticles composed of chitosan were used for intranasal delivery of antiviral siRNA to treat RSV infections by targeting the NS1 gene. The chitosan-siRNA strongly inhibited RSV replication and potentiated adaptive immune responses [251]. More recently, three siRNAs against the highly-conserved regions of SARS-CoV-2 genome were administered in vivo via lipid nanoparticles. The lipid nanoparticles-siRNA demonstrated a reduction of viral load in the lungs and enhanced survival in a mouse model [252].

### Nano-immunotherapy against viruses

Nano-based immunotherapy has gained significant attention as an effective treatment strategy for viral infections. Nevertheless, there are still challenging tasks associated with enhancing their therapeutic efficacy and minimizing side effects. Understanding the exact immune system response mechanisms against viral infection and the possible approaches to enhance immunity are the major requirements in the design of rational immunotherapy. The present section provides an overview of the immune pathways that occur in viral respiratory infections. This will be followed by a critique of the possible modulators for immune response.

Innate immune cells represent the first line of defense against viral infections. The recognition of viral pathogen-associated molecular patterns (PAMPs; nucleic acids or proteins of pathogens) by pattern recognition receptors (PRRs) stimulates the innate immune cells to release pro-inflammatory cytokines, chemokines, and/or type I IFNs that orchestrate anti-viral responses [253]. During acute viral respiratory infections, other immune cells such as alveolar macrophages, monocytes, neutrophils, and natural killer cells are activated. These activated cells produce an excessive amount of pro-inflammatory cytokines (interleukin (IL)-1β, IL-2, tumor necrosis factor (TNF)-α, IL-6, and IFN), creating a condition known as “cytokine storm” that severely impairs the respiratory epithelial cells. This phenomenon is one of the key features of SARS-CoV-2 infection but also applies to most of the major human coronavirus (SARS-CoV, MERS-CoV) and influenza A subtypes (Asian lineage avian influenza H5H1 and H7N9) [254–256].

In viral respiratory infections, dendritic cells and macrophages play a key role by orchestrating immune responses. These immune cells remove viral particles via activation of type I IFN-mediated responses and phagocytosis, with subsequent activation of the adaptive immune responses [257]. Type I IFNs block the replication of viruses by inducing the expression of IFN-stimulated genes, such as 2′-5′-oligoadenylate synthase (OAS)/RNAse L and protein kinase R (PKR) [258]. These major proteins inhibit viral proliferation through phosphorylation of eukaryotic initiation factor 2 subunit-α (eIF2α) and OAS/RNase L., resulting in impairment of the viral RNA and prevention of viral replication [259]. Type I IFNs activate effector responses mediated by the CD4^+^ and CD8^+^ T cells as well as antibody production by B lymphocytes.

According to the immune pathway, IFNα, IFNβ, and IL-6 may be suitable targets for effective immunotherapy. These molecules exert their antiviral effects by inhibiting viral replication/proliferation as well as stimulating the adaptive immune system. Most viral respiratory immunotherapy approaches utilize antiviral drugs, plasma therapy, or monoclonal neutralizing antibodies [260]. These approaches suffer from major limitations, including immune plasma availability or large-scale production of antibodies, which is expensive and time-consuming [261]. For this reason, developing advanced, rational materials is crucial for providing effective immunotherapy at a reasonable cost.

**Fig. 11:**
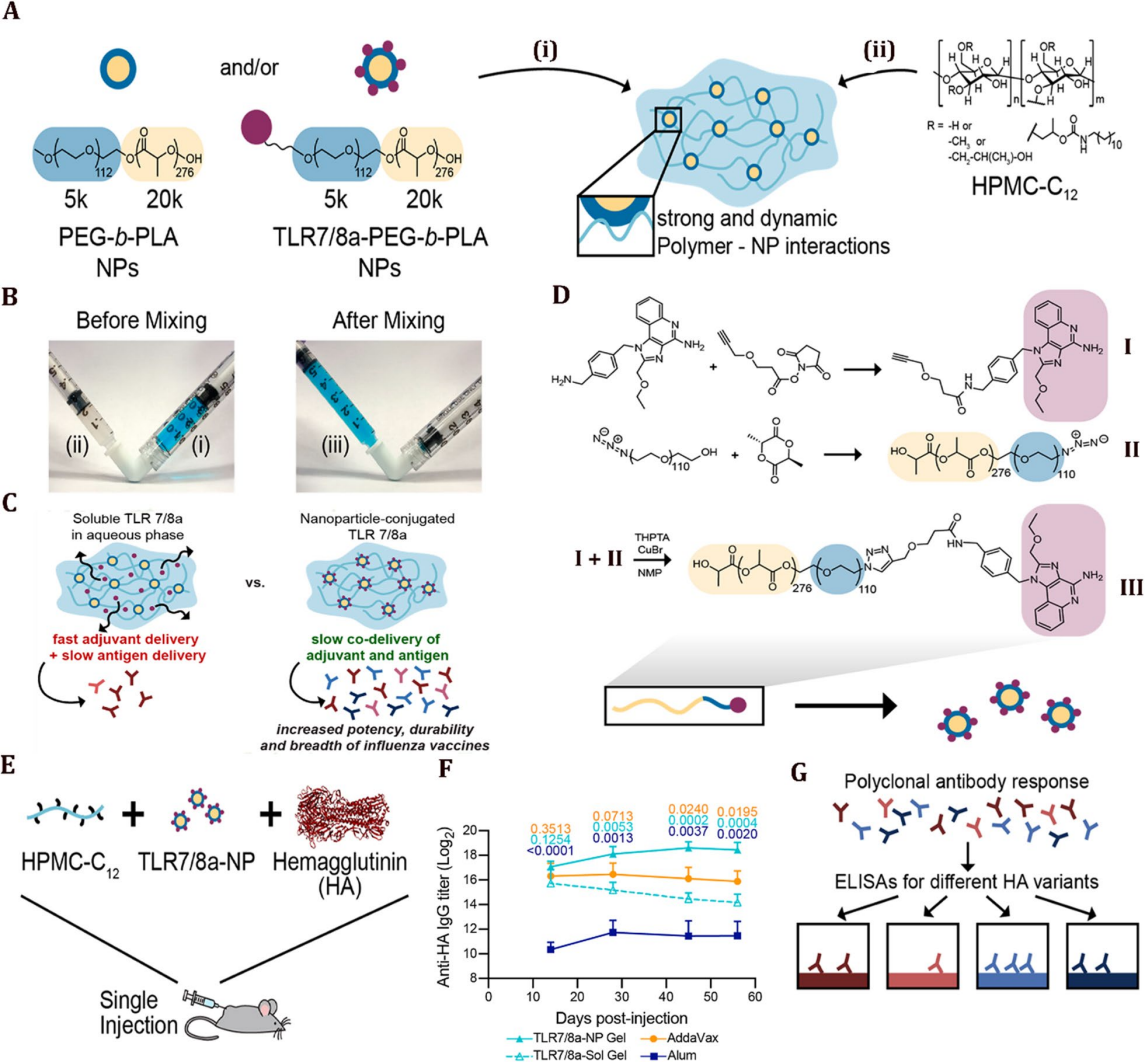
Nano-immunotherapy against respiratory viral infection. **A**, **B** Stepwise synthesis of polymer–nanoparticle (PNP) hydrogels comprising TLR7/8a-functional nanoparticles. PNP hydrogels are formed when **i** poly(ethylene glycol)-b-poly(lactic acid) (PEG-PLA) nanoparticles (NPs) or TLR7/8a-conjugated PEG-PLA NPs are combined with **ii** dodecyl-modified hydroxypropylmethylcellulose (HPMC-C12). Vaccine cargo can be added to the aqueous NP solution before mixing, which yields complete encapsulation into the fabricated hydrogels. **iii** A homogeneous gel is easily achieved using an elbow mixer or a spatula. **C** Small molecular cargo, such as TLR7/8a, can be chemically attached to the hydrogels’ PEG-PLA NP structural motif to assure long-term delivery. **D** NHS coupling of alykyne functionality to TLR7/8a (I) followed by copper-catalyzed “click” coupling to azide-terminated PEGPLA (II) yields PEG-PLA with the TLR7/8a (purple) presenting on the hydrophilic PEG (blue) terminus of the block copolymer (III). This polymer is then nanoprecipitated into the water to form TLR7/8a-functional NPs. **E** Influenza hemagglutinin subunit vaccination induces a cellular response. (a) A 2 g dosage of hemagglutinin (HA) was delivered subcutaneously in either a PNP gel prepared with 10% TLR7/8a-conjugated nanoparticles (TLR7/8a-NP Gel) and 2% HPMC-C12, a bolus of AddaVax (formulated like MF59, the most powerful adjuvant used therapeutically for influenza), or Alum. **F** Anti-HA IgG titers in serum from day 14 to day 56 after a single HA injection in TLR7/8a-NP gel, TLR7/8a-Sol gel, AddaVax bolus, or Alum bolus. P values for TLR7/8a-NP gel vs. Alum (bottom, dark blue), TLR7/8a-Sol gel (middle, light blue), and AddaVax (top, orange) (n = 4 to 5). **G** Increased breadth of antibodies toward future influenza strains (Reprinted from [270] with permission from the American Chemical Society)

Nanoparticles are excellent platforms for overcoming the restrictions associated with immunotherapy [262]. For example, nanoparticles may be used as antigen delivery systems in combination with immune agents such as antibodies to provide higher multivalent receptor cross-linking, potentiate intracellular processing, target the innate immune system, stimulate cytosolic delivery, and reduce the potential toxicity of immunomodulatory factors [263]. Different antigens may be simultaneously immobilized on the surface of the nanomaterials for more effective activation of the immune system. Hence, nanomaterials are not only delivery systems but can also act as immunomodulatory agents. The nanomaterials that have been experimentally investigated as agents for immunological applications include lipid-based materials, polymer-based materials, dendrimers, carbon nanotubes, cyclodextrin, and gold nanoparticles [264]. Inorganic nanomaterials such as silicon and gold nanoparticles can stimulate PRRs on dendritic cells to induce the expression of pro-inflammatory cytokines (e.g., IL-1, IL-6, IL-12, IFN-α, and TNF-α), as well as reduce the anti-inflammatory factors (e.g., transforming growth factor-β1 and IL-10) [265, 266]. The gold nanoparticles also stimulate T cell adaptive immune responses and promote the phagocytic activity of dendritic cells. Polyethylene glycol-conjugated IFNα and hyaluronic acid (HA)-modified gold nanoparticles (HA–gold nanoparticle/IFNα complex) have potent antiviral effects against pathogens [267]. Polymer- and lipid-based nanomaterials can induce CD8^+^/CD4^+^ T cells and increase antigen cross-presentation, which are necessary for effective immunotherapy [268, 269]. As an example, in order to extend the local codelivery of hemagglutinin and a toll-like receptor 7/8 agonist (TLR7/8a) adjuvant, a physically cross-linked polymer-nanoparticle hydrogel was manufactured recently (Fig. 11) [270]. The dynamic mesh of the polymer-nanoparticle hydrogels permits co-diffusion of the adjuvant and protein antigen (hemagglutinin) by binding the TLR7/8a to an NP motif inside the hydrogels (TLR7/8a-NP), allowing for sustained co-delivery of these two physiochemically different molecules. In comparison to clinically used adjuvants, subcutaneous distribution of polymer-nanoparticle hydrogels containing hemagglutinin and TLR7/8a-NP enhances the amplitude and duration of antibody titers in response to a single injection vaccination in mice. Furthermore, as compared to clinical vaccine adjuvants, the polymer-nanoparticle gel showed slow delivery of influenza vaccines which resulted in a greater range of antibody responses against future influenza variations [270]. Despite the advancements in the application of nanomaterials in immunotherapy, very few studies reported the use of nano-based immunotherapy for treating viral respiratory infections.

## Theranostic applications of nanostructures

**Fig. 12:**
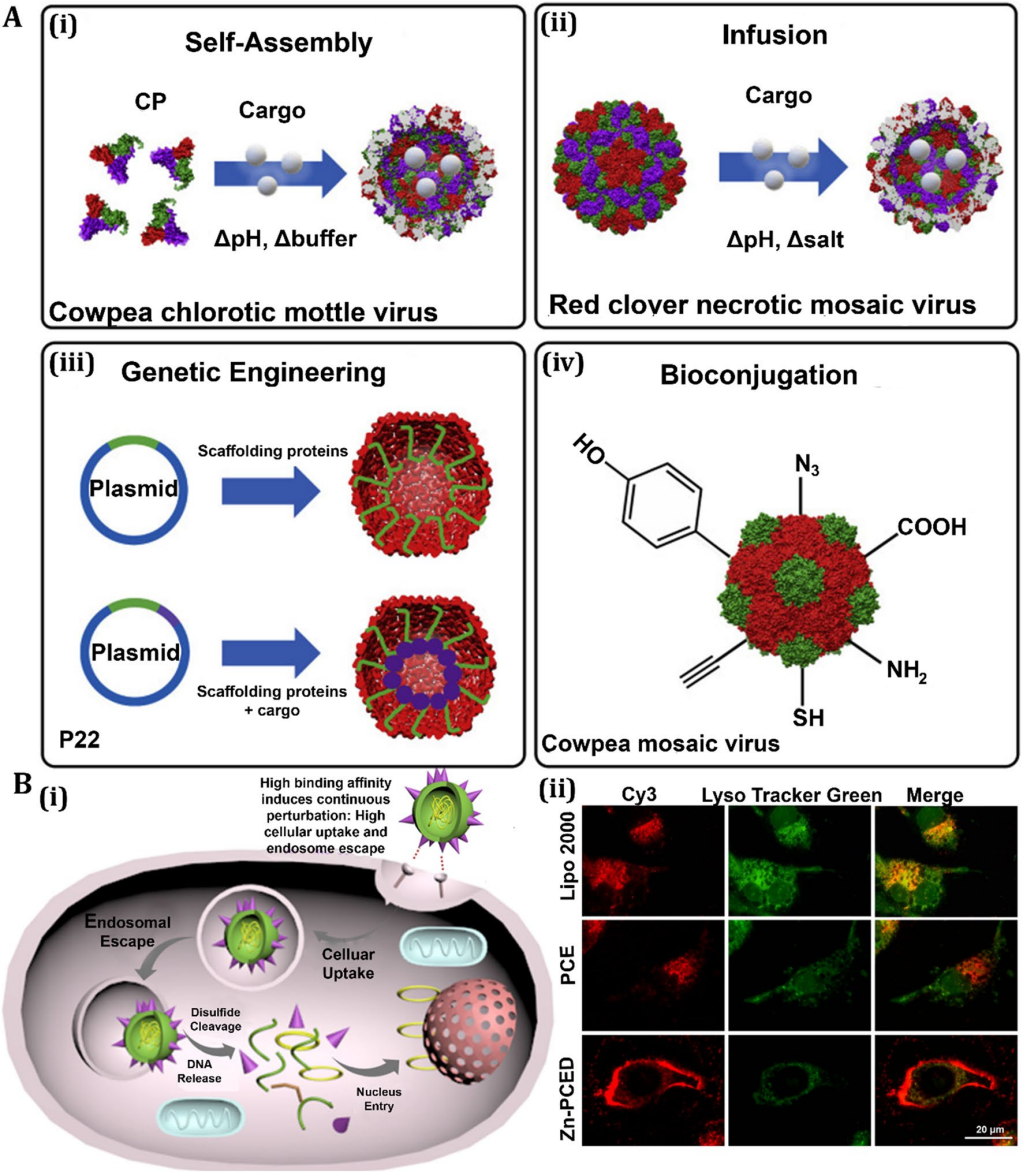
**A** Different strategies used by virus-like particles (VLPs) to carry cargo molecules: **i** self-assembly around cargo, **ii** cargo infusion, **iii** genetic engineering, and **iv** bioconjugation on VLP surface. Reprinted from [274] with permission from Elsevier. **B** The use of VLP as a gene delivery system: **i** the virus-mimicking vector Zn-PCED (containing zinc, PC1, PC2, and PC3 polymers, ε-polylysine and 2,2′-dipicolylamine) condenses DNA to form a polyplex mimicking viral structures and functions. The high affinity between the Zn coordinative residue and the cell membrane and the lipopeptide shell, facilitates cellular uptake of the polyplex, thereby contributing to high “infectivity”. DNA release is achieved by glutathione-triggered disulfide cleavage once they are relocated into the cytoplasm. **ii** Endosomal escape of PCE/Cy3-DNA and Zn-PCED/Cy3-DNA polyplexes in HeLa cells (Cy3 is a greenish-yellow fluorescent dye). Reprinted from [286] with permission from the American Chemical Society

Theranostic nanoparticles represent state-of-the-art technology for simultaneous diagnosis with the treatment of diseases [271]. The combination of treatment with imaging may be used for monitoring disease progression to confirm the effectiveness of the therapeutic method. As discussed previously, various nanocarriers, including lipid-based carriers, polymeric-based carriers, nucleic acid-based carriers, and metal-based carriers, have been developed to diagnose or treat viral infections [272]. However, agents with both diagnosis and antiviral treatment properties are required for developing nanotheranostic platforms. Apart from the previously described inorganic and organic nanoparticles, VLPs are other revolutionary platforms for the next generation of diagnostics and therapeutics. The VLPs are generally more stable compared to liposomes. They are also less toxic than metal nanoparticles and have a more uniform structure than polymer nanoparticles. VLPs are typically icosahedral or rod-shaped-like nanoparticles consisting of viral structural proteins, with a 20–200 nm size range. They self-assemble to form protein scaffolds that retain the native viral structure without containing the genetic material. Hence VLPs are incapable of replicating within host cells [273]. VLPs are flexible platforms for encapsulating and delivering a wide range of biologic and synthetic payloads, including siRNA, aptamers, proteins, peptides, antiviral drugs, and contrast agents. Different strategies have been employed for carrying cargo molecules inside and outside the capsid (Fig. 12A) [274]. They typically include four mechanisms: (i) VLP self-assembly around cargo molecule through changing buffer, pH and ionic strength in vitro (e.g., VLPs derived from cowpea chlorotic mottle viruses) [275]; (ii) infusion of the cargo molecule within the capsid of VLPs through passive diffusion (e.g. VLPs derived from red clover necrotic mosaic viruses) [276]; (iii) genetic engineering approaches using exogenous molecules that are genetically conjugated to protein scaffolds for loading therapeutics (e.g., VLPs derived from P22 phages) [277]; and (iv) bioconjugation onto virus-derived VLPs via surface modifications [278].

Most VLPs have a high immunogenic profile and can stimulate immune cells, including dendritic cells and macrophages. This advantage may be harnessed to deliver therapeutic nucleic acids to innate immune cells for the treatment of viral infection. For instance, intranasal delivery of VLPs derived from the influenza virus resulted in increases in immunity against the virus through the stimulation of innate immune responses [279]. VLPs are one of the most useful candidates for vaccine development because they induce a high level of antibodies and T-cells that result in inhibiting further infections. To date, several VLP-based vaccines have been commercially approved, such as human papillomavirus vaccines and hepatitis B vaccines. Vaccines based on VLPs are currently being developed for targeting influenza and the Epstein-Barr virus [280–284]. Another advantage of VLPs is their applicability as a vector in gene therapy. VLPs can accurately deliver a transgene to the mutation site for encoding a therapeutic protein or a DNA-repair protein with the purpose of altering gene expression [285]. For example, viral spike-mimicking nanoparticles with efficient cell binding and excellent endosomal escape capability have been developed using a Zn coordination ligand. The Zn ligand exhibited a high affinity towards phosphate-rich cell membranes to enhance transfection efficiency (Fig. 12B) [286]. VLPs are valuable theranostic platforms because they protect the nucleic acid cargoes from enzymatic degradation. These advanced VLP platforms closely resemble the structure and functions of viruses, resulting in potentially higher transfection efficacy in vitro and in vivo. These novel nanoparticles may be detected using non-invasive imaging modalities such as magnetic resonance imaging, fluorescence, and positron emission tomography [287]. VLPs offer a multivalent theranostic platform for next-generation personalized diagnosis, imaging, and therapeutics against viral infections.

Nucleic acid-based nanoparticles such as aptamers are advanced platforms that are used for both clinical diagnosis and treatment. Aptamers are short single-stranded RNA or DNA oligonucleotides with the potential capacity for detection and targeted delivery of therapeutics to destined cells, tissues, bacteria, and viruses [288]. Because aptamers bind to their specific target through a unique structural configuration, they have some advantages compared to monoclonal antibodies in terms of binding specificity, selectivity, and stability [289]. Smaller size aptamers are ideal for in vivo application, compared with antibodies. Notably, the functionalization of aptamers is not necessary for binding immunofluorescence dyes and drugs [290]. To date, aptamers have been developed for a wide range of viruses, including SARS-CoV and influenza viruses [291]. For example, RNA aptamers have been developed for targeting the NSP10 (NTPase/helicase) of SARS-CoV [292]. The aptamers efficiently bound and inhibited the DNA duplex unwinding activity of the SARS-CoV helicase. In another study, a DNA aptamer was immobilized on nitriloacetic acid magnetic beads; the assembly was bound effectively to SARS-CoV helicase, an essential enzyme for viral replication [293]. The efficacy of two types of aptamers (G-quadruplex and non-G-quadruplex) against SARS-CoV helicase was examined. The non-G376 quadruplex aptamer specifically inhibited the unwinding activity of SARS-CoV helicase [294]. Another RNA aptamer was conjugated to quantum dots so that it could be recognized by fluorescence imaging. The assembly was immobilized on a SARS-CoV nucleocapsid protein-glass chip system to specifically detect SARS-CoV nucleocapsid protein through fluorescence imaging [295]. These examples illustrate that aptamers are a resourceful multivalent theranostic platform for diagnosing and treating viral respiratory infections.

## Challenges and perspectives

After reaching control of the SARS-CoV-2 pandemic, the world will need novel methods to treat and diagnose respiratory viral infectious diseases. For example, a new method for preparing nasal swabs for collecting samples from the upper respiratory tract was recently developed using 3D printing technology [296]. Researchers are facing challenges associated with the biocompatibility of nanostructure-based preparations. The rapid rate of mutation in the virus is the most critical challenge which can reduce the usefulness of the vaccine developed against the original form of the virus or its earlier variants. For instance, using an RBD-based mRNA vaccine against SARS-CoV-2 may not be reasonable because RBD is variable in the sequence [297]. In addition, more research has to be performed to reduce the risk and side effects associated with the use of nanomaterials.

Biomaterials have been used successfully for the delivery of nucleic acids (RNA/DNA), proteins, antiviral agents, and vaccines to alleviate the COVID-19 pandemic [298]. Many areas can benefit from smart materials such as proteins, self-assembled and metallic vaccines, organoid technology, and CRISPR-based biosensors. From a diagnostic point of view, biosensors with very high precision are still required. While there has been some progress in using biomaterials to make novel biosensors and vaccines, the field is still wide open for developing therapeutics for diagnosing and treating future unforeseen coronavirus infections. Devices such as ultra-low-cost centrifuge will be beneficial for expediting the isolation and purification of viral samples derived from the saliva and blood of infected patients. Cryogenic electron microscopy and confocal laser scanning microscopy will be useful in investigating viral structure and composition. The development of an organ-on-a-chip system will be helpful for testing drugs for COVID-19 treatment. The different behavior between hosts and SARS-CoV-2 is another major challenge that requires evaluating the behavioral variability using novel organoids and organ-on-a-chip systems [299]. Adoption technologies such as robotics and microfluidics will improve drug screenings and vaccine production scalability [300].

During the pandemic, the world required a large number of nanofiber masks and personal protective equipment (PPE) [301]. With the use of novel technology such as 3D printing and nano-electrospinning, large-scale production of masks and PPE is possible. To confront future challenges, collaboration among different fields, clinicians, and industries will be required for developing novel biomaterials for the prevention, treatment, and monitoring of viral diseases [302]. The electrospinning technique was used for nanofibers production. The nanofibers have the potential for drug delivery systems and have extensive applications in the delivery of antibiotics. There are several polymers e.g., polycaprolactone, polyvinyl alcohol, polyethylene oxide, cellulose, and chitosan are employed for producing nanofibers [303]. In a study, nanofibers made from polycaprolactone and poly(lactic-co-glycolic)acid were employed for the tunable release of different anti-HIV drugs. It was expressed that the ratio of polycaprolactone and poly(lactic-co-glycolic)acid was diverse to attain continuous release ranging from 24 h to 30 days. ZnO nanorods and Ag nanoparticles have also been employed to mix antiviral properties into the electrospun polymer composites. For instance, electrospun poly 3-hydroxybutyrate-co-3-hydroxy valerate/Ag nanofiber was prepared to attain antiviral surfaces. The coating presented excellent antiviral properties and efficiency contrary to norovirus substitutes [304]. In another research, ZnO–Ag nanoparticles decorated on the poly (methyl methacrylate) nanofibers were used as defensive mats. The mats displayed excellent antiviral properties for preventing corona and influenza viruses. Furthermore, the combined photocatalyst improved the organic pollutants’ degradation, allowing self-cleaning defensive mats [305].

It should be noted that the Omicron variant of SARS-CoV-2 has a shorter incubation period (2–3 days) compared to the 5 days observed for the original virus. Clinical symptoms of the Omicron variant include runny nose, sore throat, headache, and fatigue, among others. These symptoms of the Omicron variant will disappear after several days, and there is a quick recovery. Furthermore, there is a reduced probability of hospitalization, severe disease, and death in patients infected with the Omicron variant. Nevertheless, there are challenges that need to be considered. For instance, monoclonal antibodies are effective against other variants such as the Delta variant. However, these antibodies appear to be ineffective against the Omicron variant, with the possibility of re-infection and vaccine-mediated immunity escape. Therefore, new therapeutic strategies have to be developed in the near future for the Omicron variant as well as new post-Omicron variants. There are false results associated with the use of RT-PCR for Omicron diagnosis, which pleads for the development of more advanced nanomaterial-based biosensors.

## Data Availability

Not applicable.
